# Supplementary material for: A town level comprehensive intervention study to reduce salt intake in China: protocol for a cluster randomised controlled trial
Source: BMJ Open. 2020 Jan 9;10(1):e032976. doi: 10.1136/bmjopen-2019-032976 (PMC6955555; doi:10.1136/bmjopen-2019-032976)
Supplement: Author's manuscript [file bmjopen-2019-032976.draft_revisions.pdf]

BMJ Open

BMJ Open is committed to open peer review. As part of this commitment we make the peer review history of every article we publish publicly available.

When an article is published we post the peer reviewers' comments and the authors' responses online. We also post the versions of the paper that were used during peer review. These are the versions that the peer review comments apply to.

The versions of the paper that follow are the versions that were submitted during the peer review process. They are not the versions of record or the final published versions. They should not be cited or distributed as the published version of this manuscript.

BMJ Open is an open access journal and the full, final, typeset and author-corrected version of record of the manuscript is available on our site with no access controls, subscription charges or pay-per-view fees (http://bmjopen.bmj.com).

If you have any questions on BMJ Open's open peer review process please email info.bmjopen@bmj.com

BMJ Open

A town-level comprehensive intervention to reduce salt intake in Chinese residents: A cluster randomized controlled trial

Journal:	*BMJ Open*
Manuscript ID	bmjopen-2019-032976
Article Type:	Protocol
Date Submitted by the Author:	15-Jul-2019

Complete List of Authors:	Tang, Biwei; Inner Mongolia Medical University<br>Xu, Jianwei; National Center for Chronic and Noncommunicable Disease Control and Prevention, Chinese Center for Disease Control and Prevention,<br>Liu, Min; National Center for Chronic and Noncommunicable Disease Control and Prevention, Chinese Center for Disease Control and Prevention<br>Bai, Yamin; National Center for Chronic and Noncommunicable Disease Control and Prevention, Chinese Center for Disease Control and Prevention<br>Yan, Wei; Jiangxi Provincial Center for Disease Control and Prevention<br>Zhou, Xue; Heilongjiang Provincial Center for Disease Control and Prevention<br>Xu, Zhihua; Qinghai Provincial Center for Disease Control and Prevention<br>He, Jun; Sichuan Provincial Center for Disease Control and Prevention<br>Jin, Donghui; Hunan Provincial Center for Disease Control and Prevention<br>Sun, Jixin; Hebei Provincial Center for Disease Control and Prevention<br>Li, Yuan; The George Institute for Global Health, Peking University Health Science Centre<br>Wu, Jing; National Center for Chronic and Noncommunicable Disease Control and Prevention, Chinese Center for Disease Control and Prevention<br>Zhang, Puhong ; The George Institute at Peking University Health Science Center, Diabetes Program

Keywords:	Hypertension < CARDIOLOGY, salt, clustered randomised control trial, intervention

SCHOLARONE™
Manuscripts

I, the Submitting Author has the right to grant and does grant on behalf of all authors of the Work (as defined in the below author licence), an exclusive licence and/or a non-exclusive licence for contributions from authors who are: i) UK Crown employees; ii) where BMJ has agreed a CC-BY licence shall apply, and/or iii) in accordance with the terms applicable for US Federal Government officers or employees acting as part of their official duties; on a worldwide, perpetual, irrevocable, royalty-free basis to BMJ Publishing Group Ltd ("BMJ") its licensees and where the relevant Journal is co-owned by BMJ to the co-owners of the Journal, to publish the Work in this journal and any other BMJ products and to exploit all rights, as set out in our licence.

The Submitting Author accepts and understands that any supply made under these terms is made by BMJ to the Submitting Author unless you are acting as an employee on behalf of your employer or a postgraduate student of an affiliated institution which is paying any applicable article publishing charge ("APC") for Open Access articles. Where the Submitting Author wishes to make the Work available on an Open Access basis (and intends to pay the relevant APC), the terms of reuse of such Open Access shall be governed by a Creative Commons licence – details of these licences and which Creative Commons licence will apply to this Work are set out in our licence referred to above.

Other than as permitted in any relevant BMJ Author's Self Archiving Policies, I confirm this Work has not been accepted for publication elsewhere, is not being considered for publication elsewhere and does not duplicate material already published. I confirm all authors consent to publication of this Work and authorise the granting of this licence.

For peer review only

A town-level comprehensive intervention to reduce salt intake in Chinese residents: A cluster randomized controlled trial

Biwei Tang[1], Jianwei Xu[2], Min Liu[2], Yamin Bai[2], Wei Yan[3], Xue Zhou[4], Zhihua Xu[5], Jun He[6], Donghui Jin[7], Jixin Sun[8], Yuan Li[9], Jing Wu[2], Puhong Zhang[9]

[1] Inner Mongolia Medical University, Hohhot, China

[2]National Center for Chronic and Noncommunicable Disease Control and Prevention, Chinese Center for Disease Control and Prevention, Beijing, China

[3]Jiangxi Provincial Center for Disease Control and Prevention, Nanchang, China

[4]Heilongjiang Provincial Center for Disease Control and Prevention, Harbin, China

[5]Qinghai Provincial Center for Disease Control and Prevention, Xining, China

[6]Sichuan Provincial Center for Disease Control and Prevention, Chengdu, China

[7]Hunan Provincial Center for Disease Control and Prevention, Changsha, China

[8]Hebei Provincial Center for Disease Control and Prevention, Shijiazhuang, China

[9]The George Institute for Global Health at Peking University Health Science Center, China.

Correspondence to:

Jing Wu

National Center for Chronic and Noncommunicable Disease Control and Prevention, Chinese Center for Disease Control and Prevention

No. 27, Nanwei Road

Xicheng District | Beijing, 100050 P.R. China

Tel: +86 10 8315 4663

E-mail: wujingcdc@163.com

Puhong Zhang

The George Institute for Global Health at Peking University Health Science Center,

Level 18, Tower B, Horizon Tower, No. 6 Zhichun Rd,

Haidian District | Beijing, 100088 P.R. China

Tel: +86 10 8280 0577 ext 512

For peer review only

Fax: +8610 8280 0177

E-mail: zpuhong@georgeinstitute.org.cn

A total of 3,975 words, excluding title page, abstract, references, figures and tables.

For peer review only

ABSTRACT

Introduction: High salt (sodium) intake is one of the causes of high blood pressure in the population, and hypertension is an independent risk factor for stroke and coronary heart disease. Salt reduction is a recommended measure to control a rise in high blood pressure. In response to the high salt intake, Action on Salt China(ASC) project will be implemented in China. In order to better assess the impact of salt reduction at intervention sites, salt intake will be evaluated utilizing a 24-hour urine collection method. The project's primary objective is to evaluate comprehensive salt-reduction intervention outcomes and intervention effects in town/street, and provide strong evidence for the national government to develop salt-reduction and blood-pressure lowering strategies on a large scale.

Methods and analysis: Using a clustered randomised control trial(RCT) design, 40 towns distributed in 6 different provinces were assigned to the intervention group and the control group. An initial baseline survey will be carried out among 2500 randomly selected samples from the two groups. Information of salt-related KAP, blood pressure and 24-hour urinary sodium will be collected. Under the guidance of local government and Center for Disease Control and Prevention(CDC), comprehensive intervention policies and activities will conducted at pilot sites. The control group did not take interventions. The project will conduct two rounds of assessment after 1 year and 2-3 years respectively. The primary goal is a change in the 24-hour timeframe urinary sodium levels from the baseline. The secondary goal is a change in salt-related KAP and blood pressure compared to the baseline.

Ethics and dissemination: The study has been reviewed and approved by the Institutional Review Board of the National Center for Chronic and Noncommunicable Disease Control and Prevention, the Chinese Center for Disease Control and Prevention. Results will be disseminated through presentations, publications and social media.

Trial registration number: ChiCTR1800018119

Key words: Hypertension; salt; clustered randomised control trial; intervention

For peer review only

Strengths and limitations of this study

1. Our study is extensive and involves a variety of collective units such as families, schools, units and restaurants.

2. Through this study to achieve long-term low salt intake of the population.

3. Our research uses a variety of interventions, including education, using mobile apps, conducting lectures and so on.

4. Our study can be extended to nationwide implementation in the future.

For peer review only

INTRODUCTION

Studies have shown that high salt (sodium) intake is one of the causes of high blood pressure levels and hypertension in a population, and hypertension is an independent risk factor for stroke and coronary heart disease, accounting for 62% of strokes and 49% of coronary heart disease.[1] Approximately 177 million people had hypertension in 2002 and raised BP attributed to 2.33 million cardiovascular disease(CVD) deaths in 2005.[2] Hypertension is a 'silent killer' and 75% of the Chinese hypertensive individuals are not aware that they have raised blood pressure(BP).[3] Dirtary salt intake is the major factor that increases BP and is largely responsible for the rise in BP with age.[4,5] In China, salt intake is very high with an average of 12-14g/day.[6,7] There is compelling evidence in adults that a modest reduction in salt intake lowers BP and reduces cardiovascular risk.[5,8-11] And healthcare resources are limited, a reduction in salt intake is highly cost-effective.[12,13] Salt reduction is a recommended measure to control a rise in high blood pressure. Indeed, salt reduction is one of the most cost-effective measures to prevent CVD in high-income as well as low-income and middle-income countries(LMICs).[13-15] The daily salt intake currently recommended by World Health Organization(WHO) is 5g (the Chinese government recommends 6g). According to the results of the 2002 National Nutrition Survey, salt consumption was estimated at 12 grams per day among Chinese adults, which is twice that of China's recommended dietary guideline. The World Health Organization has recommended salt reduction as one of the top three priority actions to tackle the global crisis in non-communicable disease.[12,16]

Raised blood pressure caused by excessive salt consumption is highly prevalent in China.[3] The problem is particularly marked in northern China, where salt intake is high in adults.[17] Unlike in developed countries, the major source of salt in the Chinese diet is salt added by the consumers themselves during food preparation.[18,19] In response to the high salt intake, ASC project will be implemented in China, targeting the implementation of comprehensive strategies and measures to achieve a decrease in salt intake. In order to better assess the impact of salt reduction at intervention sites, salt intake will be evaluated utilizing a 24-hour urine collection method (see above). Generally, the 24-hour urine collection method is considered as the 'gold standard' to evaluate salt intake. The assessment of sodium intake will be calculated on the basis of a 24-hour urine collection in the baseline survey, mid-term survey and final evaluation.

For peer review only

This project’s primary objective is to evaluate comprehensive salt-reduction intervention outcomes and intervention effects in town/street, and provide strong evidence for the national government to develop salt-reduction and blood-pressure lowering strategies on a large scale.

Particularly, the evaluation has the following 3 purposes: (1) To develop a comprehensive salt-reduction intervention strategy in town/street level. (2) To evaluate the effectiveness at 12 months and longer-lasting impact at 24 months on salt reduction of the intervention strategies. (3) To assess the cost-effectiveness of the intervention at 12 months and 24 months.

METHODS AND ANALYSIS

Study Setting and Overall Design

Considering multiple aspects such as geographical location, economic level and dietary habits, this project chooses to carry out on-site implementation work in six provinces including Hebei, Heilongjiang, Jiangxi, Hunan, Sichuan and Qinghai.

Using a clustered RCT design, 40 towns/streets across 6 separate provinces will be selected for the study. Two counties/districts are selected from each province. Four towns/streets are selected from each county/district. For the selection of town/streets, we will take into consideration factors of geographical area, urbanization level, number of population, structure of population, economic status, health service resource accessibility, etc. to ensure all selected towns/streets have similar characteristics. Towns/streets in the intervention group should be matched in respect to all factors with those in the control group. The inclusion criteria for towns/streets are (1) Local governments of selected counties/districts should undertake to support salt-reduction strategies; (2) Selected towns/streets should employ local government staff to carry out project-related intervention activities. We will exclude the town/streets which are involved in other salt-reduction research projects. A baseline survey will be carried out before randomization among 2500 participants recruited from the said 40 towns/streets.

All towns/streets will be allocated randomly into either intervention or control groups. Information of salt-related KAP, blood pressure and 24-hour urinary sodium levels will be collected. Comprehensive intervention strategies will be delivered to the intervention group for 12 months. Two waves of post-project evaluation will be conducted: an efficacy evaluation after 12 months, and an effectiveness evaluation after 24 months.

For peer review only - http://bmjopen.bmj.com/site/about/guidelines.xhtml

Study Population

The inclusion criteria for evaluated participants are (1) age: 18-75 years; (2) maximum of one family member per family; (3) local residents for over 6 months, no plan to relocate within 12 months; (4) agreement to sign informed consent form. The exclusion criteria for evaluated participants are (1) pregnant women and those in lactation period; (2) currently participating in any other clinical trial; (3) suffering from any severe psychiatric or physical diseases that might impact intervention and follow-up; (4) samples for 24-hour urine collection will be rejected for anyone satisfying any of the following conditions: a) urine cannot be collected due to aconuresis; b) the candidate has difficulty to collect urine and is unable to find an assistant; c) patients with acute/chronic urinary tract infection, vaginal infection and perianal infection; d) patients with acute hemorrhagic diseases in urinary tract, vagina and digestive tract; e) patients with severe vomiting and diarrheic symptoms.

Intervention

In order to promote the achievement of the goal of reducing salt, salt reduction advocacy will be delivered at national level among the control and intervention groups, the whole society is widely involved, giving full play to the role of industry associations and other social groups to promote the implementation of salt reduction measures. Regarding to the intervention in 12 months, under the technical support from local CDC, local government staff will coordinate with workers from neighbourhood offices, local women federations and local elementary/junior middle schools staff to implement comprehensive salt reduction interventions in the intervention counties (districts), targeting the major sources of salt intake in China. Including mass publicity and education, interventions by communities, schools and catering units, and salt reduction interventions based on primary care institutions.

Salt reduction publicity Each intervention county (district) mobilizes the whole society by giving full play to the advantages of the media, and carries out the theme publicity activities of the salt reduction action. Use publicity days or important holidays at least twice a year, such as *World Salt Reduction Week*, *National Nutrition Week*, etc. to carry out health knowledge promotion with the theme of salt reduction, distribute brochures and disseminate core information on salt reduction. And at least once a year to organize the county (district) participation of the masses of the "Salt and Health" cultural and publicity activities, using local mass cultural activities to select popular forms, such as knowledge contest, family

For peer review only

health gastronomy cooking contest or other activities, to create a strong action atmosphere to reduce salt. Our intervention should also include establishing a good public salt reduction environment and making more people aware of salt reduction related knowledge and skills. Through various health education materials as a carrier to promote salt and health knowledge, publicize salt reduction knowledge and skills through posters, local media or self-media such as WeChat public account to increase the coverage of publicity audiences, especially young and middle-aged occupational groups.

In the process of mass publicity, we must ensure that the form is diverse, the coverage is wide, and the content is updated in a timely manner. At the same time, various activities must strive for the publicity and coverage of the local mainstream media, and expand the influence to ensure the publicity effect of each event. And encouraged counties (districts) to fully integrate local culture and customs, innovative forms and content, and carry out mass publicity and extension work.

Salt reduction based on community All communities/villages in the towns/streets that have been intervened shall carry out salt reduction environment construction, and the core information of salt reduction shall be conveyed by putting up posters, hanging slogans and distributing pamphlets in conspicuous places. Organizing community or village family chefs and family members to conduct at least one training on salt reduction knowledge and skills (key intervention community/village at least two times) every year, or carry out other forms of community themed activities, and distribute salt limiting spoons and other intervention tools. For the community/village family chefs and family members, the family salt intake monitoring activities were carried out through the "Health Salt" WeChat small program or the salt value evaluation booklet. In the implementation of the intervention measures, we should fully investigate the enthusiasm of community workers, and develop grassroots health instructors, so that the relationship between staff and the masses will be closer, more conducive to the salt reduction intervention work in the community.

Salt reduction based on school In all the schools in the intervention town, publicity posters should be put up in the publicity boards or school canteens. And the school recess broadcast can be used to publicize the knowledge of salt reduction, so as to create a good campus environment for salt reduction. Salt and health training activities are conducted at least once a year, using opportunities such as centralized teacher training or school parent-teacher

For peer review only

meetings. Or in combination with local conditions, carry out "AppSalt use activities", "previous section of salt reduction health education class", "watch a salt reduction science animation", "salt reduction handwritten newspaper" and other forms of school salt reduction publicity activities. In addition, we also need to actively promote the use of AppSalt by students' parents in combination with local conditions, by means of training or school WeChat group, or organize a class to carry out the use of AppSalt, so that families can reduce the use of AppSalt under the support of AppSalt.

Salt reduction based on restaurant Salt reduction activities can be carried out in restaurants with a certain scale, and can be combined with the healthy canteen and health work established in the demonstration area. The knowledge of salt reduction can be publicized through posters, table decorations, and accessible brochures, so as to create a restaurant environment conducive to salt reduction. In the restaurant and the canteen, we will combine with the competent departments of catering units to train the catering chef at least once a year, and provide technical training for the restaurant and restaurant chefs to guide them to cook different salty dishes. When the conditions are ripe, the restaurant will launch a meal-reducing salt reduction service promotion campaign in the restaurant, and we will choose to carry out the meal reduction label in individual catering units.

Primary care institutions reduce salt This project also covers the community health service center or village clinic under the jurisdiction of the township/street. The project also covers the community health service centers/village clinics under the township/street jurisdiction, and publicizes salt reduction knowledge in the form of posters, accessible brochures or banners in the township/street jurisdiction and community health service centers/village clinics where the intervention is carried out. Conditional health agencies can broadcast salt reduction publicity videos to create a salt reduction environment in primary health care institutions through the above methods. County (district) centers for disease control and prevention carry out training for relevant personnel of township health centers and community health service centers/village health offices under the jurisdiction of the intervention at least twice a year, focusing on the training of salt reduction knowledge, and relying on the national basic public health service projects, training salt reduction guidelines for patients with hypertension. All primary health care institutions should hold lectures and guidance on salt reduction at least twice a year. Community health service centers/village clinics use daily outpatient clinics or organize lectures to promote knowledge about salt

For peer review only

reduction and prevention of hypertension, and provide guidance on salt reduction for patients with hypertension.

No additional intervention on salt reduction will be conducted among the control group in Year 1. In Year 2, intervention strategies will be made available to the general public, including those originally randomized into the control group. All participants in this study will be encouraged to follow salt-reduction strategies.

Control sites: No salt-reduction intervention is conducted in the control group.

Sample size

Our aim is to reduce sodium intake by 25 mmo/L. According to the study design, a sample of 20 cluster pairs (40 clusters) with 50 individuals per cluster achieves 80% power to detect a difference of 25 between the group means when the standard deviation is 85 and the within-pair coefficient of variation between clusters is 0.080. A two-sided T-test of the mean difference is assumed, with a significance level of 0.05. Thus a total of 2500 individuals are required for each wave of evaluation, and 1250 individuals for each group.

Outcome measures

The primary outcome is the decrease of 25 mmol/L in the 24-hour urinary sodium level from the baseline. The secondary outcome is the change in salt-related KAP and blood pressure from the baseline.

Outcome assessments

The project will conduct a baseline survey before the intervention, conduct a mid-term assessment after 1 year of intervention, and conduct a terminal survey after the intervention. Both the intervention and the control county/district carried out the same assessment at the same time. Our baseline survey included questionnaires, physical measurements, and 24-hour urine collection.

The questionnaire survey conducted a face-to-face inquiry survey by trained and qualified investigators through the mobile electronic data collection system. According to the content and sequence of the questionnaire, the basic information of the respondents and relevant

For peer review only

behavioral risk factors, knowledge, attitudes and behaviors related to salt reduction and prevention and control of hypertension, hypertension and related expenses were collected.

Physical measurements are accurately measured by trained researchers using calibrated measuring instruments, including height, weight, waist circumference, blood pressure, and heart rate.

During the 24-hour urine collection, we asked the respondents to empty the bladder, record the start time, dispense the urine collection equipment, and inform the respondents of the urine retention precautions, and specify the collection time for the next day. When retrieving the urine collection equipment, we need to ask for the last urination time. If the respondent does not have the last urinary or urine, the last urine should be collected on site and the end time recorded. When investigating urine, the investigator should also judge whether the urine is qualified. If the urine is unqualified, it is necessary to re-schedule the collection. Finally, the qualified urine is sealed and transported to the laboratory for unified testing. The test indexes included urinary sodium, urinary potassium, urinary creatinine and urinary microalbumin.

DATA COLLECTION AND ANALYSIS

Data collection methods

CRFs will be designed to collect data of both the baseline survey and evaluation, including demographic characteristics, salt-related KAP, blood pressure, and 24-hour urinary sodium levels. Besides such data collected at the baseline, participants lost and reasons for loss need to be recorded both at the stages of 12-month follow-up and longer-lasting effectiveness evaluation at 24 months. Electronic data capture technology will be employed to support data collection.

Data management

The data of this survey was collected through the mobile electronic data acquisition system (EDC), which was developed by Beijing University of Aeronautics and Astronautics. Relevant data such as questionnaires, physical measurements, urine collection and intervention processes were collected by the EDC system.

Statistical Analysis

For peer review only

The effectiveness of the intervention package on primary and secondary outcomes will be evaluated at 12 months after the first-year intervention. The difference in 24-hour sodium excretion levels as well as the secondary outcomes will be compared between the two groups using linear mixed models, with participants nested within family units and families nested within community/village units. We will include group (intervention, control), time (baseline, end of trial), and time×group interaction, with the time×group interaction term indicating differential change by group from baseline to the end of the trial. To account for missing data on continuous outcomes, we will use the likelihood based random effects model that uses all available data and provides valid estimates of the intervention effects when data are missing at random. We will adjust for the stratification variables at randomization and potential confounding variables. We will also carry out various sensitivity analyses to examine the robustness of the conclusions of the primary analysis.

When evaluating the longer-lasting effectiveness of the intervention package at 24 months, the same methods as mentioned above will be applied, with replacing the major/secondary outcomes at 12 months by those at 24 months. We assume the effect of the scale-up will be offset between the intervention and control group.

SAS will be used for the analyses. Results will be reported as mean, SD, SE, and 95% confidence interval when appropriate. All analyses are two sided, and $P<0.05$ is considered significant.

Process evaluation

In order to ensure that the interventions can be implemented according to the plan and requirements, the project will carry out process evaluation, strengthen process management. Process evaluation includes evaluation of the two parts of the indicator system and method. We need to establish a good working system, develop an effective intervention plan, and strictly control the implementation process of the intervention. Each intervention county (district) shall formulate an intervention plan, and report the progress of the intervention on a quarterly basis according to the activity plan. The report includes the content of the activity, the time of the event, the implementer, the participants, and other relevant documents, photos and objects and so on. The national and provincial supervision teams went to the intervention site to check the construction of the salt reduction environment, check the posting and placement of posters, brochures and other promotional materials, and understand the

For peer review only

distribution of materials, lectures and training. Using semi-structured interviews, interview with key informants such as project staff, managers, and intervention personnel in the county (district) to understand the completion of interventions, the effects of interventions, and problems in interventions.

Project status and timelines

Preparations are made from April to August 2018. The baseline assessment was conducted from September to October 2018. Since two rounds of effect evaluations are to be carried out after 12 months and 24 months, a mid-term evaluation will be conducted between August and September 2019, and the final follow-up evaluation will be conducted from August to September 2019.

ETHICS AND DISSEMINATION

Research Ethics Approval and Consent

The study has been reviewed and approved by the Institutional Review Board of the National Center for Chronic and Noncommunicable Disease Control and Prevention, the Chinese Center for Disease Control and Prevention. Written consent will be obtained from all participants who will be free to discontinue their participation at any time, with no explanation required.

Consent or Assent

Consent for participation in the project will be sought both at the cluster level and the individual level. Cluster level consent of the community will be obtained through a consultation process involving government (at provincial, county and township levels) and village leaders. The project, including the process of random assignment of communities to intervention and control conditions and the nature of interventions, will be explained at a face-to-face meeting. Questions will be answered and all relevant stakeholder groups invited to consult with their members and reflect upon the project. Individual consent for participation in outcome surveys will be obtained from all persons selected in a standard manner via provision of a participant information sheet, explanation and discussion as required, and the collection of written consent from those willing to take part.

Written consent will be obtained from all participants who will be free to discontinue their

For peer review only

participation at any time, with no explanation required.

Acknowledgements

The author would like to thank street/community residents, catering companies and primary care staff for their opinions on the development of the intervention program.

Contributors

PZ and JW conceived the project. JX, ML, YB, YL, JW and PZ participated in the design and implementation of the project. All authors facilitates Patient and Public Involvement and were responsible for setting up the study in each site. BT and JX wrote the first draft of the manuscript, they contributed equally to this paper. All authors contributed to the refinement of the study protocol and approved the final manuscript.

Funding

This work is supported by the National Institute of Health Research using Official Development Assistance (ODA) funding (16/136/77).

Competing interests

No competing interests.

Dissemination policy

The findings of this study will be disseminated through discussion or presentations at selected conferences, peer-reviewed publications and the general media.

For peer review only

REFERENCES

1 World Health Organization. Reducing risks, promoting healthy life. Geneva, Switzerland: World Health Organisation, 2002. http://www.who.int/whr/2002 (Accessed 13 Jul 2019).
2 He J, Gu D, Chen J, et al. Premature deaths attributable to blood pressure in China: a prospective cohort study. *Lancet* 2009, 374:1765-1772.
3 Wu Y, Huxiey R, Li L, et al. Prevalence, awareness, treatment, and control of hypertension in China: data from the China National Nutrition and Health Survey 2002. *Circulation* 2008, 118:2679-2686.
4 Intersalt Cooperative Research Group. Intersalt: an international study of electrolyte excretion and blood pressure. Results for 24hour urinary sodium and potassium excretion.

BMJ 1988, 297:319-328.
5 He FJ, MacGregor GA. Reducing population salt intake worldwide: from evidence to implementation. *Prog Cardiovasc Dis* 2010, 52:363-382.
6 Zhou BF, Stamler J, Dennis B, et al.Nutrient intakes of middle-aged men and women in China, Japan, United Kingdom, and United States in the late 1990s:the INTERMAP study. *J Hum Hypertens* 2003, 17:623-630.
7 He FJ, Wu Y, Feng XX, et al. School based education programme to reduce salt intake in children and their families (School-EduSalt): cluster randomised controlled trial. *BMJ* 2015, 350:h770
8 He FJ, Li J, Macgregor GA. Effect of longer term modest salt reduction on blood pressure: cochrane systematic review and meta-analysis of randomised trials. *BMJ* 2013, 346: f1325.
9 Aburto NJ, Ziolkovska A, Hooper L, et al. Effect of lower sodium intake on health: systematic review and meta-analyses. *BMJ* 2013, 346: f1326.
10 He FJ, MacGregor GA. Salt reduction lowers cardiovascular risk: meta-analysis of outcome trials. *Lancet* 2011, 378:380-382.
11 He FJ, Campbell NRC, Ma Y, MacGregor GA, et al. Errors in estimating usual sodium intake by the Kawasaki formula alter its relationship with mortality: implications for public health. *Int J Epidemiol* 2018, 47(6):1784-1795.
12 Beaglehole R, Bonlta R, Horton R, et al. Priority actions for the non-communicable disease crisis. *Lancet.* 2011, 377:1438-1447.
13 Asaria P, Chisholm D, Mathers C, et al. Chronic disease prevention: health effects and financial costs of strategies to reduce salt intake and control tobacco use. *Lancet* 2007, 370:2044-2053.
14 Bibbins-Domingo K, Chertow GM, Coxson PG, et al. Projected effect of dietary salt reductions on future cardiovascular disease. *N Engl J Med* 2010, 362:590-599.
15 National Institute for Health and Clinical Excellence(NICE). Guidance on the prevention of cardiovascular disease at the population level. http://guidance.nice.org.uk/PH25 (Accessed 13 Jul 2019).
16 World Health Organization. First global ministerial conference on healthy lifestyles and noncommunicable disease control. https://www.who.int/nmh/events/moscow_ncds_2011/en/ (Accessed 13 Jul 2019).
17 Zhao L, Stamler J. Yan LL, et al. Blood pressure differences between northern and southern Chinese: role of dietary factors: the international Study on Macronutrients and Blood Pressure. *Hypertension* 2004, 43:1332-1337.
18 Anderson CA, Appel LJ, Okuda N, et al. Dietary sources of sodium in China, Japan, the United Kingdom, and the Untied States, women and men aged 49 to 59 years: the INTERMAP study. *J AM Diet Assoc* 2010, 110:736-745.
19 Xu J, Wang M, Chen Y, et al. Estimation of salt intake by 24-hour urinary sodium excretion: a cross-sectional study in Yantai, China. *BMC Public Health* 2014, 14:136.

For peer review only

BMJ Open

A town level comprehensive intervention study (CIS) to reduce salt intake in Chinese residents: Protocol of a cluster randomized controlled trial

Journal:	*BMJ Open*
Manuscript ID	bmjopen-2019-032976.R1
Article Type:	Protocol
Date Submitted by the Author:	10-Oct-2019
Complete List of Authors:	Xu, Jianwei; National Center for Chronic and Noncommunicable Disease Control and Prevention, Chinese Center for Disease Control and Prevention,<br>Tang, Biwei; National Center for Chronic and Noncommunicable Disease Control and Prevention, Chinese Center for Disease Control and Prevention; Inner Mongolia Medical University<br>Liu, Min; National Center for Chronic and Noncommunicable Disease Control and Prevention, Chinese Center for Disease Control and Prevention<br>Bai, Yamin; National Center for Chronic and Noncommunicable Disease Control and Prevention, Chinese Center for Disease Control and Prevention<br>Yan, Wei; Jiangxi Provincial Center for Disease Control and Prevention<br>Zhou, Xue; Heilongjiang Provincial Center for Disease Control and Prevention<br>Xu, Zhihua; Qinghai Provincial Center for Disease Control and Prevention<br>He, Jun; Sichuan Provincial Center for Disease Control and Prevention<br>Jin, Donghui; Hunan Provincial Center for Disease Control and Prevention<br>Sun, Jixin; Hebei Provincial Center for Disease Control and Prevention<br>Li, Yuan; The George Institute for Global Health, Peking University Health Science Centre<br>He, Feng; Queen Mary University of London, Wolfson Institute of Preventive Medicine<br>MacGregor, Graham; Queen Mary University of London, Wolfson Institute of Preventive Medicine<br>Wu, Jing; National Center for Chronic and Noncommunicable Disease Control and Prevention, Chinese Center for Disease Control and Prevention<br>Zhang, Puhong ; The George Institute at Peking University Health Science Center, Diabetes Program
**Primary Subject Heading**:	Epidemiology
Secondary Subject Heading:	Epidemiology, Public health
Keywords:	Hypertension < CARDIOLOGY, EPIDEMIOLOGY, STATISTICS & RESEARCH METHODS, PUBLIC HEALTH

SCHOLARONE™
Manuscripts

I, the Submitting Author has the right to grant and does grant on behalf of all authors of the Work (as defined in the below author licence), an exclusive licence and/or a non-exclusive licence for contributions from authors who are: i) UK Crown employees; ii) where BMJ has agreed a CC-BY licence shall apply, and/or iii) in accordance with the terms applicable for US Federal Government officers or employees acting as part of their official duties; on a worldwide, perpetual, irrevocable, royalty-free basis to BMJ Publishing Group Ltd ("BMJ") its licensees and where the relevant Journal is co-owned by BMJ to the co-owners of the Journal, to publish the Work in this journal and any other BMJ products and to exploit all rights, as set out in our licence.

The Submitting Author accepts and understands that any supply made under these terms is made by BMJ to the Submitting Author unless you are acting as an employee on behalf of your employer or a postgraduate student of an affiliated institution which is paying any applicable article publishing charge ("APC") for Open Access articles. Where the Submitting Author wishes to make the Work available on an Open Access basis (and intends to pay the relevant APC), the terms of reuse of such Open Access shall be governed by a Creative Commons licence – details of these licences and which Creative Commons licence will apply to this Work are set out in our licence referred to above.

Other than as permitted in any relevant BMJ Author's Self Archiving Policies, I confirm this Work has not been accepted for publication elsewhere, is not being considered for publication elsewhere and does not duplicate material already published. I confirm all authors consent to publication of this Work and authorise the granting of this licence.

For peer review only

A town level comprehensive intervention study (CIS) to reduce salt intake in Chinese residents: Protocol of a cluster randomized controlled trial

Jianwei Xu[1], Biwei Tang[1,2], Min Liu[1], Yamin Bai[1], Wei Yan[3], Xue Zhou[4], Zhihua Xu[5], Jun He[6], Donghui Jin[7], Jixin Sun[8], Yuan Li[9,10], Feng J He[11], Graham A MacGregor[11], Jing Wu[1], Puhong Zhang[9,10]

[1]National Center for Chronic and Noncommunicable Disease Control and Prevention, Chinese Center for Disease Control and Prevention, Beijing, China

[2]Inner Mongolia Medical University, Hohhot, China

[3]Jiangxi Provincial Center for Disease Control and Prevention, Nanchang, China

[4]Heilongjiang Provincial Center for Disease Control and Prevention, Harbin, China

[5]Qinghai Provincial Center for Disease Control and Prevention, Xining, China

[6]Sichuan Provincial Center for Disease Control and Prevention, Chengdu, China

[7]Hunan Provincial Center for Disease Control and Prevention, Changsha, China

[8]Hebei Provincial Center for Disease Control and Prevention, Shijiazhuang, China

[9]The George Institute for Global Health at Peking University Health Science Center, China.

[10]Faculty of Medicine, University of New South Wales, Australia.

[11]Wolfson Institute of Preventive Medicine, Barts and The London School of Medicine & Dentistry, Queen Mary University of London, UK

Correspondence to:

Jing Wu

National Center for Chronic and Noncommunicable Disease Control and Prevention, Chinese Center for Disease Control and Prevention

For peer review only

No. 27, Nanwei Road

Xicheng District | Beijing, 100050 P.R. China

Tel: +86 10 8315 4663

E-mail: wujingcdc@163.com

Puhong Zhang

The George Institute for Global Health at Peking University Health Science Center,

Level 18, Tower B, Horizon Tower, No. 6 Zhichun Rd,

Haidian District | Beijing, 100088 P.R. China

Tel: +86 10 8280 0577 ext 512

Fax: +8610 8280 0177

E-mail: zpuhong@georgeinstitute.org.cn

A total of 5416 words, excluding title page, abstract, references, figures and tables.

For peer review only

ABSTRACT

Introduction: Salt intake in China (≈12g/d) twice the recommended upper limit (5g/d) by World Health Organization. In response to the high salt intake, Action on Salt China (ASC) was launched in 2017. As one of four randomized controlled trials (RCTs) in ASC, a comprehensive intervention study (CIS) was designed to test if all the components of interventions adopted by other RCTs are acceptable, scalable and effective if provided to a region in real world..

Methods and analysis: Using a cluster RCT design, 2688 participants sampled from 48 towns (cluster) of 12 counties in 6 provinces were assigned to the intervention group and the control group. Randomization was done after baseline survey completed within each country. Information of salt-related knowledge, attitude and practice (KAP), blood pressure and 24-hour urinary sodium were collected. The intervention in CIS covered government engagement, health education and all the intervention components targeting restaurants, house cook, and primary school students and their families used in other parallel RCTs. The control group did not receive the intervention package. The project will be followed up for two years, with only the first year is covered by intervention. The primary outcome is salt intake measured by 24-hour urinary sodium excretion after one year. The secondary outcomes is the long lasting effectiveness on salt reduction measured by the same method, as well as salt-related KAP and blood pressure after one and two years follow-up. Process evaluation and health economics analysis will be conducted as well.

Ethics and dissemination: The study has been reviewed and approved by the Institutional Review Board of the National Center for Chronic and Noncommunicable Disease Control and Prevention, the Chinese Center for Disease Control and Prevention, and Queen Mary Research Ethics Committee. Results will be disseminated through presentations, publications and social media.

Trial registration number: ChiCTR1800018119

Key words: Hypertension; salt; clustered randomised control trial; intervention

For peer review only

Strengths and limitations of this study

1. This is a cluster randomized controlled trial to test a complex intervention on salt reduction in various settings in China.

2. 24-hour urine sodium excretion is used for primary outcome evaluation.

3. Adaption to the intervention according to regional context and resources is allowed, but need to be recorded and reported.

4. Process monitoring during project implementation and process evaluation at the end of intervention will be done to supervise the fidelity and adaption to the intervention, and understand whether, why and how the interventions work in the study.

5. The intervention delivery is led by county government, potential contamination may exist for participants in towns of control group under the same county.

For peer review only

INTRODUCTION

Studies have shown that high salt (sodium) intake is one of the causes of high blood pressure levels and hypertension in a population, and hypertension is an independent risk factor for stroke and coronary heart disease, accounting for 62% of strokes and 49% of coronary heart disease.[1] Approximately 177 million people had hypertension in 2002 and raised blood pressure (BP) attributed to 2.33 million cardiovascular disease (CVD) deaths in 2005.[2] Hypertension is a 'silent killer' and 75% of the Chinese hypertensive individuals are not aware that they have BP.[3] Dietary salt intake is the major factor that increases BP and is largely responsible for the rise in BP with age.[4,5] In China, salt intake is very high with an average of 12-14g/day.[6,7] There is compelling evidence in adults that a modest reduction in salt intake lowers BP and reduces cardiovascular risk.[5,8-11] Salt reduction is one of the most cost-effective measures to prevent CVD in high-income as well as low-income and middle-income countries (LMICs).[12,13] The daily salt intake currently recommended by World Health Organization (WHO) is 5g (the Chinese government recommends 6g). According to the results of the 2002 National Nutrition Survey, Chinese adults' daily salt intake is twice that of China's recommended dietary guidelines. The World Health Organization has recommended salt reduction as one of the top three priority actions to tackle the global crisis in non-communicable disease.[14]

Unlike in developed countries, the major source of salt in the Chinese diet is salt added by the consumers themselves during food preparation.[15,16] In response to the high salt intake, Action on Salt China (ASC) unit was set up in 2017 with the purpose of reducing salt intake by implementing a comprehensive national salt reduction programme.[17] ASC is funded by the National Institute for Health Research (NIHR) of UK, led by Queen Mary University of London, The George Institute China, Chinese Center for Disease Control and Prevention (China CDC), and several other key agencies in China. Under ASC unit. A series of programmes has been designed and are being implemented, including campaigns for health education and pre-packaged food salt reduction as well as four cluster randomized controlled trials (RCT) to

For peer review only

develop and test specific intervention packages targeting different settings or populations. The four RCTs are (1) Application-based Intervention Study (AIS) targeting schoolchildren and their families;[18] (2) Housewife-based Intervention study (HIS) supporting using less salt in home cooking; (3) Restaurant-based Intervention Study (RIS), targeting salt intake from restaurants; and (4) Comprehensive Intervention Study (CIS) as a scale-up study, i.e. the study we introduce in this paper.

CIS was designed to simulate real world scale up, in which all the available interventions adopted by other RCTs were provided to the government of participating counties. The purpose of the study is to evaluate the acceptability for each components of intervention, the effectiveness (including cost-effectiveness) of the intervention right after a one year intervention, the long-lasting effectiveness after the intervention stopped for one year, and finally provide evidence for national scale-up. This paper describes the specific study design, implementation and current status of CIS.

METHODS AND ANALYSIS

Study Setting and Overall Design

Considering multiple aspects such as geographical location, economic level and dietary habits, this project chooses to carry out in six provinces including Hebei, Heilongjiang, Jiangxi, Hunan, Sichuan and Qinghai, covering north and south, east and west, and middle China.

CIS is designed as a cluster RCT, already launched since September 2018 and to be completed by the end of 2020. The clusters are 48 towns (named street in urban areas, but only “town” is used hereafter for simplification) selected from 12 counties (named district in urban areas, but only “county” is used hereafter) across the 6 provinces. Each province selects 2 project counties mainly from rural or suburb areas where the livings of local residents are more independent of surroundings when compared with those in central urban area. This may help to minimize contamination among counties and towns. In each county, 4 towns, with similar population and economic development level, and not adjacent to each other, are selected with the purpose of avoiding imbalance on

For peer review only

potential confounders between intervention and control groups due to the small number (48) of clusters and contamination of intervention to control group. 56 eligible participants are selected from each town to receive randomized treatment, intervention or not, and evaluation during a two years follow-up. The 4 towns are evenly randomized into the intervention or control group right after the baseline survey has been completed for all the four towns within a county. The randomization methodology is acknowledged in advance to all the investigators at provincial, county and township levels, but the result of randomization keeps concealed until the centralized randomization finished.

The implementation of intervention is led by county investigators with the support from local governments, but the delivery of the intervention package is conducted at township level or the cluster level. To avoid contamination, all the intervention activities are required to be conducted within the intervention town. The intervention will be provided for the first 12 months. The effectiveness of intervention will be evaluated right after completion of the 12 months intervention (mid-term assessment) and after another 12 months follow-up to find its long-lasting effectiveness (endpoint assessment).

Study Population and participant recruitment

The target population is all adult residents in study sites. In this study, the inclusion criteria for evaluated participants are (1) age: 18-75 years; (2) maximum of one family member per family; (3) local residents for over 6 months, with no plan to move within 24 months; (4) agreement to sign informed consent form. The exclusion criteria for evaluated participants are (1) pregnant women and those in lactation period; (2) currently participating in any other clinical trial; (3) suffering from any severe psychiatric or physical diseases that might impact intervention and follow-up; (4) unable or not suitable to collect 24-hour urine due to the following conditions: a) aconuresis; b) acute/chronic urinary tract infection, vaginal infection and perianal infection; c) acute hemorrhagic diseases in urinary tract, vagina and digestive tract; d) severe vomiting and diarrheic symptoms; e) other conditions difficult for urine collect

For peer review only

and unable to find an assistant.

A two-stage sampling is conducted to recruit eligible participants. Firstly, two villages (named committees in urban areas) are randomly selected, and then 28 eligible participants are randomly selected from each village, i.e. 56 participants for each town. The procedure of village and participant selection is conducted by county investigators with the support of a specially designed smartphone application. To fulfil the random selection, the names of villages as well as the names of residents in the selected villages need to be uploaded to the server through the app, and a centralized randomization result will be presented through the app to the county investigators. The reasons why not eligible for some residents are also recorded through the app.

Intervention

In order to promote all intervention components for salt reduction, a multi-section engagement strategy is recommended to local governments at county, township and village levels. The government agencies and other major stakeholders to be engaged also include local centers for disease control and prevention (CDC), women federations, propaganda centers, hospitals, schools, restaurants, supermarkets, etc.. The county CDCs will lead and coordinate the implementation of the comprehensive salt reduction interventions, including mass publicity and education, interventions by communities, schools and catering units, and salt reduction interventions based on primary care institutions. The overall health promotion and the major interventions targeting different populations or settings within the intervention towns are summarized below.

Salt reduction publicity within the intervention towns Each county mobilizes the whole township society in intervention group by carrying out various theme publicity activities for salt reduction. These include (1) to carry out health knowledge promotion by training and distributing brochures and disseminate core information on salt reduction leveraging publicity days or important holidays at least twice a year, such as *World Salt Reduction Week*, *National Nutrition Week*, etc.; (2) at least once a year to organize a mass cultural and publicity activities on "Salt and Health", using local

For peer review only

popular forms, such as knowledge contest, family health gastronomy cooking contest or other activities, to create a strong action atmosphere to reduce salt; (3) to establish a good public salt reduction environment such as healthy park, healthy road, healthy oil station, etc.; and (4) to promote salt and health knowledge and skills through posters and self-media such as WeChat public account to increase the coverage of publicity audiences, especially young and middle-aged occupational groups.

In the process of mass publicity, we encourage to leverage the local culture and customs, use innovative forms and content, and encourage that the form is diverse, the coverage is wide, and the content is updated in a timely manner. At the same time, contamination to the control towns must be avoided by limiting the activities within the intervention towns.

Salt reduction based on community All communities/villages in the intervention towns try to establish salt reduction environment by putting up posters, hanging slogans and distributing pamphlets in conspicuous places. Organizing community or village family chefs and family members to conduct at least one training on salt reduction knowledge and skills every year, or carry out other forms of community themed activities, and distribute salt limiting spoons and other intervention tools. For the community/village family chefs and family members, the family salt intake monitoring activities carry out through the "Health Salt" WeChat applet or the salt intake evaluation booklet. Eight standard, no more than 2 minutes, audios are provided for loudspeaker broadcasting for all villages in the intervention towns.

Salt reduction based on school In all the schools in the intervention town, publicity posters should be put up in the publicity boards or school canteens. And the school recess broadcast can be used to publicize the knowledge of salt reduction, so as to create a good campus environment for salt reduction. Salt and health training activities are conducted at least once a year, using opportunities such as centralized teacher training or school parent-teacher meetings. Or in combination with local conditions, carry out "AppSalt use activities" (AppSalt is an app-based platform designated for salt reduction through primary school, which is the key intervention in AIS[18]) "previous section of

salt reduction health education class", "watch a salt reduction science animation", "salt reduction handwritten newspaper" and other forms of school salt reduction publicity activities. In addition, we also need to actively promote the use of AppSalt by students' parents in combination with local conditions, by means of training or school WeChat group, or organize a class to carry out the use of AppSalt.

Salt reduction based on restaurant Salt reduction activities can be carried out in restaurants with a certain scale, and can be combined with the healthy canteen and health workplace. The knowledge of salt reduction can be publicized through posters, table decorations, and accessible brochures, so as to create a restaurant environment conducive to salt reduction. In the restaurant and the canteen, we will combine with the competent departments of catering units to train the catering chef at least once a year, and provide technical training for the restaurant and restaurant chefs to guide them to cook different salty dishes. When the conditions are ripe, the restaurant will launch a meal-reducing salt reduction service promotion campaign in the restaurant, and we will choose to carry out the meal reduction label in individual catering units.

Salt reduction based on primary care service This project also covers the community health service under the jurisdiction of town (township hospitals) and village (village clinics) by publicizing salt reduction knowledge in the form of posters, accessible brochures or banners in the hospitals and clinics. If available, these facilities can broadcast salt reduction publicity videos. The county CDCs carry out training at least twice a year for all the care providers under the jurisdiction of the intervention towns, by combining with the routine training of national basic public health service. All primary health care institutions should hold lectures and guidance on salt reduction at least twice a year. Primary care providers should deliver salt reduction knowledge and skills during the routine outpatient clinics to promote salt reduction and prevention of hypertension for the visiting patients.

Theoretically, as mentioned above, although some salt reduction activities are mandatory, it is acceptable for the local governments to select some of the intervention tools or materials and add some if they think reasonable. But the degree, coverage and

For peer review only

cost must be recorded during the project implementation. No additional intervention on salt reduction will be conducted among the control group in Year 1. In Year 2, intervention strategies will be made available to the general public as a national scale-up, including those originally randomized into the control group. The hypotheses is that the national scale-up has the same impact to the two groups, and the difference on 24-hour urinary sodium excretion at the end of year 2 can reflex the pure long-lasting effectiveness of the intervention conducted in year 1.

Sample size

Referring to our unpublished before-after effectiveness of sodium reduction by 37 mmol/L in our recently completed Shandong salt reduction,[19] our aim is to reduce sodium intake by at least 25 mmol/d (1.46 g/d salt) from baseline with comparison to the control group. According to the research design, we randomly select 2688 eligible participants from 48 towns (56 each) in 6 provinces. Assuming the maximum drop rate is 20% for towns (from 48 to 40) and 10% for participants (from 56 to 50) within the two years follow-up, this sample size and sampling method would have a 80% power to detect a difference of 25 mmol/d between the group means assuming the standard deviation is 85.0 mmol/d and intraclass correlation coefficient (ICC) is 0.080, with a two-sided analyses and a significance level of 0.05. The parameter estimations for effectiveness, standard deviation and ICC are also based on Shandong study, and also similar to the results in a cluster RCT conducted in China.[7]

Outcomes and outcome assessment

The primary outcome is sodium intake measured by 24-hour urinary sodium exertion. The secondary outcomes include the change in salt-related knowledge, attitude and practice (KAP), and blood pressure.

The project will conduct a baseline survey before randomization, a mid-term assessment after 1 year of intervention, and an endpoint assessment two years later from baseline. Both the intervention and the control towns will carry out the same

For peer review only

assessments in parallel. Our baseline survey included questionnaires, physical measurements, and 24-hour urine collection.

The questionnaire survey conducted a face-to-face inquiry survey by trained and qualified investigators through a mobile electronic data collection system.[20] According to the content and sequence of the questionnaire, the basic information of the respondents and relevant behavioral risk factors, knowledge, attitudes and behaviors related to salt reduction and prevention and control of hypertension, hypertension and related expenses were collected. Physical measurements are accurately measured by trained researchers using calibrated measuring instruments, including height, weight, waist circumference, blood pressure, and heart rate.

During the 24-hour urine collection, we asked the respondents to empty the bladder, record the start time, dispense the urine collection equipment, and inform the respondents of the urine retention precautions, and specify the collection time for the next day. When retrieving the urine collection equipment, we need to ask for the last urination time. If the respondent does not remember the time of the last urine collection, then the final urine should be collected on site and the end time recorded. If any of the following three problems occur, it is determined that the urine sample is unacceptable: (1) forget to collect or splash urine more than 10% of the total; (2) urine is contaminated with blood, stool or other impurities; (3) excessive sweating, diarrhea or vomiting during collection. If any of the above situations is reported, another 24-hour urine collection should be re-scheduled. Finally, the qualified urine is sealed and transported to the laboratory for unified testing. The test indexes included urinary sodium, urinary potassium, urinary creatinine and urinary microalbumin.

The staff participating in the field investigation are the backbone of the project counties. We will conduct unified training for them and conduct assessment. Only those who pass the assessment can conduct the field investigation. And they will be compensated accordingly.

For peer review only

DATA COLLECTION AND ANALYSIS

Data collection methods

Case report forms (CRFs) will be designed to collect data of both the baseline survey and evaluation, including demographic characteristics, salt-related KAP, blood pressure, and 24-hour urinary sodium levels. Besides such data collected at the baseline, participants lost and reasons for loss need to be recorded both at the stages of 12-month follow-up and longer-lasting effectiveness evaluation at 24 months. Electronic data capture technology will be employed to support data collection.

Data management

The data of this survey was collected through the mobile electronic data acquisition system (mEDC),[20] which was developed by Beijing University of Aeronautics and Astronautics. Relevant data such as questionnaires, physical measurements, urine collection and intervention processes were collected by the mEDC system.

Statistical Analysis

The effectiveness of the intervention package on primary and secondary outcomes will be evaluated at 12 months after one-year intervention, and at 24 months after the intervention stopped for one year. The linear mixed models will be used to model both outcome measures at 12 months (primary analysis) and 24 months, with adjustment for the baseline value (same as the analysed outcome), and with participants nested within village units. The group difference will be estimated at each time point using the time point × group as interaction term. The town (cluster variable at the level of randomization) and the repeated measures at the participant level will be treated as random effects. To account for missing data on continuous outcomes, we will use the likelihood based random effects model that uses all available data to provide valid estimates of the intervention effects when data are missing at random. We will also carry out various sensitivity analyses to examine the robustness of the conclusions of the primary analysis.

For peer review only

When evaluating the longer-lasting effectiveness of the intervention package at 24 months, the same methods as mentioned above will be applied, with replacing the major/secondary outcomes at 12 months by those at 24 months. We assume the effect of the scale-up will be offset between the intervention and control group.

SAS will be used for the analyses. Results will be reported as mean, SD, SE, and 95% confidence interval when appropriate. All analyses are two sided, and $P<0.05$ is considered significant.

Process monitoring and evaluation

In order to ensure to supervise the fidelity and adaption to the intervention, process monitoring will be carried out throughout the intervention period of time. Process monitoring includes evaluation of indicator system and method. For each county, governance, working system, and effective intervention plan must be ready before the initiation of intervention. During the implementation, all the intervention will be recorded and reported on a quarterly basis according to the activity plan. The report includes the content of the activity, the time of the event, the implementer, the participants, and other relevant documents, photos and objects and so on. The national and provincial supervision teams will visit the intervention sites quarterly to check the construction of the salt reduction environment, check the posting and placement of posters, brochures and other promotional materials, and understand the distribution of materials, lectures and training.

At the end of year 1 and year 2, a systematic semi-structured interviews will be conducted separately to evaluate whether, why and how the specific interventions work in different settings, with the purpose of promoting the scale-up for effective salt reduction strategies and measures in China and worldwide. Key informants such as project staff, managers, and intervention personnel in village, town and county levels will be interviewed.

Economic evaluation

For peer review only

Economic evaluations will be carried out from health sector perspective to compare the comprehensive intervention with usual care, and it will entail two components: a trial-based economic evaluation and a modelled economic evaluation of long-term costs and outcomes. Intervention costs will include those in delivering the intervention which shall be recorded by the county investigators, but exclude any research and development costs. The trial-based economic evaluation will be assessed initially in terms of incremental cost per unit reduction in salt intake and systolic BP. The modelled economic evaluation with discounting will examine the cost, survival, quality of life over lifetime, via capturing various health states (including death and CVD events) to estimate incremental cost per life year saved and cost per Quality-Adjusted Life Year gained. The transition probabilities across health states and costs attached to various health states will be based on literature and the long-term effects of the reduction in salt intake or systolic BP will be derived from the trial findings and/or literature of disease progression. Sensitivity analyses will be carried out to estimate uncertainty about the primary findings associated with varying key parameters.

Project status and timelines

Preparations are made from April 2017 to August 2018. The baseline assessment was initiated from September to December 2018. 2688 eligible participants have been successfully recruited from 48 towns (4 of them located in urban areas) of 12 counties (2 of them located in urban areas) and completed the baseline survey. Since two rounds of effect evaluations are to be carried out after 12 months and 24 months, a mid-term evaluation will be conducted by the end of 2019, and the final follow-up evaluation will be conducted in December 2019.

Patient and Public Involvement

According to the actual situation in the locality, the investigation site adopts various forms to carry out propaganda and mobilization work, and introduces the meaning and purpose of the investigation to the residents. Rely on the leadership and support of the local government and grassroots organizations, master the situation, make appointments, and strive to understand, support and cooperate with the respondents.

For peer review only

After determining the identity of the investigator, we will sign an informed consent form with the investigator, then conduct a questionnaire survey, body measurements, and urine collection.

ETHICS AND DISSEMINATION

Research Ethics Approval and Consent

The study has been reviewed and approved by the Institutional Review Board of the National Center for Chronic and Noncommunicable Disease Control and Prevention, the Chinese Center for Disease Control and Prevention (201807), and Queen Mary Research Ethics Committee. Written consent will be obtained from all participants who will be free to discontinue their participation at any time, with no explanation required.

Consent or Assent

Consent for participation in the project will be sought both at the cluster level and the individual level. Cluster level consent of the community will be obtained through a consultation process involving government (at provincial, county and town levels) and village leaders. The project, including the process of random assignment of communities to intervention and control conditions and the nature of interventions, will be explained at a face-to-face meeting. Questions will be answered and all relevant stakeholder groups invited to consult with their members and reflect upon the project. Individual consent for participation in outcome surveys will be obtained from all persons selected in a standard manner via provision of a participant information sheet, explanation and discussion as required, and the collection of written consent from those willing to take part.

Written consent will be obtained from all participants who will be free to discontinue their participation at any time, with no explanation required.

Acknowledgements

The author would like to thank street/community residents, catering companies, school

For peer review only

teachers, parents of primary school students, primary care staff, and village, town and county leaders for their opinions on the development of the intervention program.

Contributors

PZ and JW conceived the project. JX, ML, YB, YL, JW, FJH, GAM and PZ participated in the design and implementation of the project. JX, BT, ML, YB, WY, XZ, ZX, JH, DJ, JS, YL, JW, FJH, GAM and PZ facilitates Patient and Public Involvement and were responsible for setting up the study in each site. BT and JX wrote the first draft of the manuscript, they contributed equally to this paper. All authors contributed to the refinement of the study protocol and approved the final manuscript.

Funding

This work is supported by the National Institute of Health Research (NIHR) (NIHR Global Health Research Unit Action on Salt China at Queen Mary University of London) using Official Development Assistance (ODA) funding (16/136/77). The views expressed in this publication are those of the author(s) and not necessarily those of the NIHR or the Department of Health and Social Care.

Competing interests

FJH is a member of the Consensus Action on Salt & Health (CASH) group, a non-profit charitable organisation, and its international branch World Action on Salt & Health (WASH) and does not receive any financial support from CASH or WASH. GAM is the Chairman of Blood Pressure UK (BPUK), Chairman of CASH and Chairman of WASH and does not receive any financial support from any of these organisations. BPUK, CASH and WASH are non-profit charitable organisations. All other authors have no competing interest to declare.

Dissemination policy

The findings of this study will be disseminated through discussion or presentations at selected conferences, peer-reviewed publications and the general media.

For peer review only

REFERENCES

1 World Health Organization. Reducing risks, promoting healthy life. Geneva, Switzerland: World Health Organisation, 2002. http://www.who.int/whr/2002 (Accessed 13 Jul 2019).

2 He J, Gu D, Chen J, et al. Premature deaths attributable to blood pressure in China: a prospective cohort study. *Lancet* 2009, 374:1765-1772.

3 Wu Y, Huxiey R, Li L, et al. Prevalence, awareness, treatment, and control of hypertension in China: data from the China National Nutrition and Health Survey 2002. *Circulation* 2008, 118:2679-2686.

4 Intersalt Cooperative Research Group. Intersalt: an international study of electrolyte excretion and blood pressure. Results for 24hour urinary sodium and potassium excretion. *BMJ* 1988, 297:319-328.

5 He FJ, MacGregor GA. Reducing population salt intake worldwide: from evidence to implementation. *Prog Cardiovasc Dis* 2010, 52:363-382.

6 Zhou BF, Stamler J, Dennis B, et al.Nutrient intakes of middle-aged men and women in China, Japan, United Kingdom, and United States in the late 1990s:the INTERMAP study. *J Hum Hypertens* 2003, 17:623-630.

7 He FJ, Wu Y, Feng XX, et al. School based education programme to reduce salt intake in children and their families (School-EduSalt): cluster randomised controlled trial. *BMJ* 2015, 350:h770

8 He FJ, Li J, Macgregor GA. Effect of longer term modest salt reduction on blood pressure: cochrane systematic review and meta-analysis of randomised trials. *BMJ* 2013, 346: f1325.

9 Aburto NJ, Ziolkovska A, Hooper L, et al. Effect of lower sodium intake on health: systematic review and meta-analyses. *BMJ* 2013, 346: f1326.

10 He FJ, MacGregor GA. Salt reduction lowers cardiovascular risk: meta-analysis of outcome trials. *Lancet* 2011, 378:380-382.

11 He FJ, Campbell NRC, Ma Y, MacGregor GA, et al. Errors in estimating usual sodium intake by the Kawasaki formula alter its relationship with mortality: implications for public health. *Int J Epidemiol* 2018, 47(6):1784-1795.

12 Bibbins-Domingo K, Chertow GM, Coxson PG, et al. Projected effect of dietary salt reductions on future cardiovascular disease. *N Engl J Med* 2010, 362:590-599.

13 National Institute for Health and Clinical Excellence(NICE). Guidance on the prevention of cardiovascular disease at the population level. http://guidance.nice.org.uk/PH25 (Accessed 13 Jul 2019).

14 World Health Organization. First global ministerial conference on healthy lifestyles and noncommunicable disease control. https://www.who.int/nmh/events/moscow_ncds_2011/en/ (Accessed 13 Jul 2019).

15 Anderson CA, Appel LJ, Okuda N, et al. Dietary sources of sodium in China, Japan, the United Kingdom, and the Untied States, women and men aged 49 to 59 years: the INTERMAP study. *J AM Diet Assoc* 2010, 110:736-745.

For peer review only

16 Xu J, Wang M, Chen Y, et al. Estimation of salt intake by 24-hour urinary sodium excretion: a cross-sectional study in Yantai, China. *BMC Public Health* 2014, 14:136.

17 He FJ, Zhang PH, Li Y, et al. Action on Salt China. *Lancet* 2018,392(10141):7-9.

18 He FJ, Zhang PH, Luo R, et al. An Application-based programme to reinforce and maintain lower salt intake (AppSalt) in schoolchildren and their families in China. *BMJ Open* 2019,9(7): e027793.

19 Chen X, Guo X, Ma J, et al. Urinary sodium or potassium excretion and blood pressure in adults of Shandong province, China: preliminary results of the SMASH project. *J Am Soc Hypertens* 2015, 9(10): 754-762.

20 Zhang J, Sun L, Liu Y, et al. Mobile Device-Based Electronic Data Capture System Used in a Clinical Randomized Controlled Trial: Advantages and Challenges. *J Med Internet Res* 2017, 19(3):e66.

For peer review only

SPIRIT

STANDARD PROTOCOL ITEMS: RECOMMENDATIONS FOR INTERVENTIONAL TRIALS

SPIRIT 2013 Checklist: Recommended items to address in a clinical trial protocol and related documents*

Section/item	Item No	Description
**Administrative information**		
Title	1	Descriptive title identifying the study design, population, interventions, and, if applicable, trial acronym(line 5-6 in page2)
Trial registration	2a	Trial identifier and registry name. If not yet registered, name of intended registry(line 57 in page 4)
	2b	All items from the World Health Organization Trial Registration Data Set(None)
Protocol version	3	Date and version identifier(None)
Funding	4	Sources and types of financial, material, and other support(line 23-34 in page 18)
Roles and responsibilities	5a	Names, affiliations, and roles of protocol contributors(line 9-48 in page 2 and line 11-21 in page 18)
	5b	Name and contact information for the trial sponsor(line 17-35 in page3)
	5c	Role of study sponsor and funders, if any, in study design; collection, management, analysis, and interpretation of data; writing of the report; and the decision to submit the report for publication, including whether they will have ultimate authority over any of these activities(line 24-30 in page14)
	5d	Composition, roles, and responsibilities of the coordinating centre, steering committee, endpoint adjudication committee, data management team, and other individuals or groups overseeing the trial, if applicable (see Item 21a for data monitoring committee)(line 21-43 in page 15)
**Introduction**		
Background and rationale	6a	Description of research question and justification for undertaking the trial, including summary of relevant studies (published and unpublished) examining benefits and harms for each intervention(line 6 in page 6)

For peer review only

	6b	Explanation for choice of comparators(None)
Objectives	7	Specific objectives or hypotheses(line 20 in page 7)
Trial design	8	Description of trial design including type of trial (eg, parallel group, crossover, factorial, single group), allocation ratio, and framework (eg, superiority, equivalence, noninferiority, exploratory)(line 33 in page 7)

Methods: Participants, interventions, and outcomes

Study setting	9	Description of study settings (eg, community clinic, academic hospital) and list of countries where data will be collected. Reference to where list of study sites can be obtained(line 37 in page 7)
Eligibility criteria	10	Inclusion and exclusion criteria for participants. If applicable, eligibility criteria for study centres and individuals who will perform the interventions (eg, surgeons, psychotherapists)(line 40 in page 8)
Interventions	11a	Interventions for each group with sufficient detail to allow replication, including how and when they will be administered(line24 in page 9)
	11b	Criteria for discontinuing or modifying allocated interventions for a given trial participant (eg, drug dose change in response to harms, participant request, or improving/worsening disease)(None)
	11c	Strategies to improve adherence to intervention protocols, and any procedures for monitoring adherence (eg, drug tablet return, laboratory tests)(None)
	11d	Relevant concomitant care and interventions that are permitted or prohibited during the trial(None)
Outcomes	12	Primary, secondary, and other outcomes, including the specific measurement variable (eg, systolic blood pressure), analysis metric (eg, change from baseline, final value, time to event), method of aggregation (eg, median, proportion), and time point for each outcome. Explanation of the clinical relevance of chosen efficacy and harm outcomes is strongly recommended(line 44 in page 12)
Participant timeline	13	Time schedule of enrolment, interventions (including any run-ins and washouts), assessments, and visits for participants. A schematic diagram is highly recommended (see Figure)(line 33 in page 16)
Sample size	14	Estimated number of participants needed to achieve study objectives and how it was determined, including clinical and statistical assumptions supporting any sample size calculations(line 18 in page 12)
Recruitment	15	Strategies for achieving adequate participant enrolment to reach target sample size(line 49 in page 16)

Methods: Assignment of interventions (for controlled trials)

For peer review only - http://bmjopen.bmj.com/site/about/guidelines.xhtml

Allocation:

Sequence generation	16a	Method of generating the allocation sequence (eg, computer-generated random numbers), and list of any factors for stratification. To reduce predictability of a random sequence, details of any planned restriction (eg, blocking) should be provided in a separate document that is unavailable to those who enrol participants or assign interventions(line 44 in page 7)
Allocation concealment mechanism	16b	Mechanism of implementing the allocation sequence (eg, central telephone; sequentially numbered, opaque, sealed envelopes), describing any steps to conceal the sequence until interventions are assigned(none)
Implementation	16c	Who will generate the allocation sequence, who will enrol participants, and who will assign participants to interventions(line 50 in page 13)
Blinding (masking)	17a	Who will be blinded after assignment to interventions (eg, trial participants, care providers, outcome assessors, data analysts), and how(none)
	17b	If blinded, circumstances under which unblinding is permissible, and procedure for revealing a participant's allocated intervention during the trial(none)

Methods: Data collection, management, and analysis

Data collection methods	18a	Plans for assessment and collection of outcome, baseline, and other trial data, including any related processes to promote data quality (eg, duplicate measurements, training of assessors) and a description of study instruments (eg, questionnaires, laboratory tests) along with their reliability and validity, if known. Reference to where data collection forms can be found, if not in the protocol(line 6 in page 14)
	18b	Plans to promote participant retention and complete follow-up, including list of any outcome data to be collected for participants who discontinue or deviate from intervention protocols
Data management	19	Plans for data entry, coding, security, and storage, including any related processes to promote data quality (eg, double data entry; range checks for data values). Reference to where details of data management procedures can be found, if not in the protocol(line 22 in page 14)
Statistical methods	20a	Statistical methods for analysing primary and secondary outcomes. Reference to where other details of the statistical analysis plan can be found, if not in the protocol(line 33 in page 14)
	20b	Methods for any additional analyses (eg, subgroup and adjusted analyses)(line 55 in page 14)

For peer review only - http://bmjopen.bmj.com/site/about/guidelines.xhtml

	20c	Definition of analysis population relating to protocol non-adherence (eg, as randomised analysis), and any statistical methods to handle missing data (eg, multiple imputation)(none)
**Methods: Monitoring**		
Data monitoring	21a	Composition of data monitoring committee (DMC); summary of its role and reporting structure; statement of whether it is independent from the sponsor and competing interests; and reference to where further details about its charter can be found, if not in the protocol. Alternatively, an explanation of why a DMC is not needed(line 23 in page 15)
	21b	Description of any interim analyses and stopping guidelines, including who will have access to these interim results and make the final decision to terminate the trial(line 45 in page 15)
Harms	22	Plans for collecting, assessing, reporting, and managing solicited and spontaneously reported adverse events and other unintended effects of trial interventions or trial conduct(none)
Auditing	23	Frequency and procedures for auditing trial conduct, if any, and whether the process will be independent from investigators and the sponsor(line 35 in page 15)
**Ethics and dissemination**		
Research ethics approval	24	Plans for seeking research ethics committee/institutional review board (REC/IRB) approval(line 16 in page 17)
Protocol amendments	25	Plans for communicating important protocol modifications (eg, changes to eligibility criteria, outcomes, analyses) to relevant parties (eg, investigators, REC/IRBs, trial participants, trial registries, journals, regulators)(none)
Consent or assent	26a	Who will obtain informed consent or assent from potential trial participants or authorised surrogates, and how (see Item 32)(line 29 in page 17)
	26b	Additional consent provisions for collection and use of participant data and biological specimens in ancillary studies, if applicable(none)
Confidentiality	27	How personal information about potential and enrolled participants will be collected, shared, and maintained in order to protect confidentiality before, during, and after the trial(line 39 in 17)
Declaration of interests	28	Financial and other competing interests for principal investigators for the overall trial and each study site(line 37 in page 18)
Access to data	29	Statement of who will have access to the final trial dataset, and disclosure of contractual agreements that limit such access for investigators

review only

For peer review only - http://bmjopen.bmj.com/site/about/guidelines.xhtml

Ancillary and post-trial care	30	Provisions, if any, for ancillary and post-trial care, and for compensation to those who suffer harm from trial participation(none)
Dissemination policy	31a	Plans for investigators and sponsor to communicate trial results to participants, healthcare professionals, the public, and other relevant groups (eg, via publication, reporting in results databases, or other data sharing arrangements), including any publication restrictions(line 51 in page 18)
	31b	Authorship eligibility guidelines and any intended use of professional writers(none)
	31c	Plans, if any, for granting public access to the full protocol, participant-level dataset, and statistical code(none)
**Appendices**		
Informed consent materials	32	Model consent form and other related documentation given to participants and authorised surrogates(line 51 in page 17)
Biological specimens	33	Plans for collection, laboratory evaluation, and storage of biological specimens for genetic or molecular analysis in the current trial and for future use in ancillary studies, if applicable(line 24 in page 13)

*It is strongly recommended that this checklist be read in conjunction with the SPIRIT 2013 Explanation & Elaboration for important clarification on the items. Amendments to the protocol should be tracked and dated. The SPIRIT checklist is copyrighted by the SPIRIT Group under the Creative Commons "Attribution-NonCommercial-NoDerivs 3.0 Unported" license.

For peer review only

BMJ Open

A town level comprehensive intervention study (CIS) to reduce salt intake in China: Protocol of a cluster randomized controlled trial

Journal:	*BMJ Open*
Manuscript ID	bmjopen-2019-032976.R2
Article Type:	Protocol
Date Submitted by the Author:	21-Nov-2019
Complete List of Authors:	Xu, Jianwei; National Center for Chronic and Noncommunicable Disease Control and Prevention, Chinese Center for Disease Control and Prevention,<br>Tang, Biwei; National Center for Chronic and Noncommunicable Disease Control and Prevention, Chinese Center for Disease Control and Prevention; Inner Mongolia Medical University<br>Liu, Min; National Center for Chronic and Noncommunicable Disease Control and Prevention, Chinese Center for Disease Control and Prevention<br>Bai, Yamin; National Center for Chronic and Noncommunicable Disease Control and Prevention, Chinese Center for Disease Control and Prevention<br>Yan, Wei; Jiangxi Provincial Center for Disease Control and Prevention<br>Zhou, Xue; Heilongjiang Provincial Center for Disease Control and Prevention<br>Xu, Zhihua; Qinghai Provincial Center for Disease Control and Prevention<br>He, Jun; Sichuan Provincial Center for Disease Control and Prevention<br>Jin, Donghui; Hunan Provincial Center for Disease Control and Prevention<br>Sun, Jixin; Hebei Provincial Center for Disease Control and Prevention<br>Li, Yuan; The George Institute for Global Health, Peking University Health Science Centre<br>He, Feng; Queen Mary University of London, Wolfson Institute of Preventive Medicine<br>MacGregor, Graham; Queen Mary University of London, Wolfson Institute of Preventive Medicine<br>Wu, Jing; National Center for Chronic and Noncommunicable Disease Control and Prevention, Chinese Center for Disease Control and Prevention<br>Zhang, Puhong ; The George Institute at Peking University Health Science Center, Diabetes Program
**Primary Subject Heading**:	Epidemiology
Secondary Subject Heading:	Epidemiology, Public health
Keywords:	Hypertension < CARDIOLOGY, EPIDEMIOLOGY, STATISTICS & RESEARCH METHODS, PUBLIC HEALTH

I, the Submitting Author has the right to grant and does grant on behalf of all authors of the Work (as defined in the below author licence), an exclusive licence and/or a non-exclusive licence for contributions from authors who are: i) UK Crown employees; ii) where BMJ has agreed a CC-BY licence shall apply, and/or iii) in accordance with the terms applicable for US Federal Government officers or employees acting as part of their official duties; on a worldwide, perpetual, irrevocable, royalty-free basis to BMJ Publishing Group Ltd ("BMJ") its licensees and where the relevant Journal is co-owned by BMJ to the co-owners of the Journal, to publish the Work in this journal and any other BMJ products and to exploit all rights, as set out in our licence.

The Submitting Author accepts and understands that any supply made under these terms is made by BMJ to the Submitting Author unless you are acting as an employee on behalf of your employer or a postgraduate student of an affiliated institution which is paying any applicable article publishing charge ("APC") for Open Access articles. Where the Submitting Author wishes to make the Work available on an Open Access basis (and intends to pay the relevant APC), the terms of reuse of such Open Access shall be governed by a Creative Commons licence – details of these licences and which Creative Commons licence will apply to this Work are set out in our licence referred to above.

Other than as permitted in any relevant BMJ Author's Self Archiving Policies, I confirm this Work has not been accepted for publication elsewhere, is not being considered for publication elsewhere and does not duplicate material already published. I confirm all authors consent to publication of this Work and authorise the granting of this licence.

For peer review only

A town level comprehensive intervention study (CIS) to reduce salt intake in China: Protocol of a cluster randomized controlled trial

Jianwei Xu[1], Biwei Tang[1,2], Min Liu[1], Yamin Bai[1], Wei Yan[3], Xue Zhou[4], Zhihua Xu[5], Jun He[6], Donghui Jin[7], Jixin Sun[8], Yuan Li[9,10], Feng J He[11], Graham A MacGregor[11], Jing Wu[1], Puhong Zhang[9,10]

[1]National Center for Chronic and Noncommunicable Disease Control and Prevention, Chinese Center for Disease Control and Prevention, Beijing, China

[2]Inner Mongolia Medical University, Hohhot, China

[3]Jiangxi Provincial Center for Disease Control and Prevention, Nanchang, China

[4]Heilongjiang Provincial Center for Disease Control and Prevention, Harbin, China

[5]Qinghai Provincial Center for Disease Control and Prevention, Xining, China

[6]Sichuan Provincial Center for Disease Control and Prevention, Chengdu, China

[7]Hunan Provincial Center for Disease Control and Prevention, Changsha, China

[8]Hebei Provincial Center for Disease Control and Prevention, Shijiazhuang, China

[9]The George Institute for Global Health at Peking University Health Science Center, China.

[10]Faculty of Medicine, University of New South Wales, Australia.

[11]Wolfson Institute of Preventive Medicine, Barts and The London School of Medicine & Dentistry, Queen Mary University of London, UK

Correspondence to:

Jing Wu

National Center for Chronic and Noncommunicable Disease Control and Prevention, Chinese Center for Disease Control and Prevention

For peer review only

No. 27, Nanwei Road

Xicheng District | Beijing, 100050 P.R. China

Tel: +86 10 8315 4663

E-mail: wujingcdc@163.com

Puhong Zhang

The George Institute for Global Health at Peking University Health Science Center,

Level 18, Tower B, Horizon Tower, No. 6 Zhichun Rd,

Haidian District | Beijing, 100088 P.R. China

Tel: +86 10 8280 0577 ext 512

Fax: +8610 8280 0177

E-mail: zpuhong@georgeinstitute.org.cn

A total of 4897 words, excluding title page, abstract, references, figures and tables.

For peer review only

ABSTRACT

Introduction: Salt intake in China (≈12g/d) is more than twice the recommended upper limit (5g/d) by World Health Organization. To reduce salt intake, Action on Salt China (ASC) was launched in 2017. As one of four randomized controlled trials (RCTs) in ASC programme, a comprehensive intervention study (CIS) was designed to test if all the components of interventions adopted by other RCTs are acceptable, scalable and effective if provided to a region in real world.

Methods and analysis: Using a cluster RCT design, 2688 participants sampled from 48 towns (cluster) of 12 counties in 6 provinces were assigned to the intervention group or the control group. Randomization was done after baseline survey has completed. Information of salt-related knowledge, attitude and practice (KAP), blood pressure and 24-hour urinary sodium were collected. The intervention includes government engagement, health education and all other intervention components targeting restaurants, home cooks, and primary school students and their families used in other RCTs. The control group did not receive the intervention package. The project will be followed up for two years, with intervention being carried out for the first year only. The primary outcome is salt intake measured by 24-hour urinary sodium excretion after one year. The secondary outcomes are the long lasting effectiveness on salt intake and blood pressure measured by the same method, as well as salt-related KAP and blood pressure after one and two years follow-up. Process evaluation and health economics analysis will be conducted as well.

Ethics and dissemination: The study has been reviewed and approved by the Institutional Review Board of the National Center for Chronic and Noncommunicable Disease Control and Prevention, the Chinese Center for Disease Control and Prevention, and Queen Mary Research Ethics Committee. Results will be disseminated through presentations, publications and social media.

Trial registration number: ChiCTR1800018119

Key words: Hypertension; salt; clustered randomised control trial; intervention

For peer review only

Strengths and limitations of this study

1. This is a cluster randomized controlled trial evaluating a complex intervention on salt reduction with 24-hour urine sodium excretion as primary outcome among 2688 participants from 48 towns (cluster) in 6 provinces in China.

2. The intervention is delivered at township level. Potential contamination may exist because the participants in control group may visit people or eat at restaurants in intervention towns.

3. As a regional complex salt reduction programme, it is hard to deliver a fixed intervention in a mandatory manner. Instead, adaption according to regional context and resources is allowed.

4. Process monitoring during project implementation and process evaluation at the end of intervention will be done to supervise the fidelity and adaption to the intervention, to understand whether, why and how the interventions work in the study.

For peer review only

INTRODUCTION

Studies have shown that high salt (sodium) intake is one of the causes of high blood pressure levels and hypertension in a population, and hypertension is an independent risk factor for stroke and coronary heart disease, accounting for 62% of strokes and 49% of coronary heart disease.[1,2,3] In China, approximately 177 million people had hypertension in 2002 and raised blood pressure (BP) attributed to 2.33 million cardiovascular disease (CVD) deaths in 2005.[4] Hypertension is a 'silent killer' and 75% of the Chinese hypertensive individuals are not aware that they have BP.[5] In China, salt intake is very high with an average of 12-14g/day.[6,7] There is compelling evidence in adults that a modest reduction in salt intake lowers BP and reduces cardiovascular risk.[3,8-11] Salt reduction is one of the most cost-effective measures to prevent CVD in high-income as well as low-income and middle-income countries.[12,13] The daily salt intake currently recommended by World Health Organization (WHO) is 5g (the Chinese government recommends 5g). According to the results of the 2002 National Nutrition Survey, Chinese adults' daily salt intake is twice that of China's recommended dietary guidelines. The World Health Organization has recommended salt reduction as one of the top three priority actions to tackle the global crisis in non-communicable disease.[14]

Unlike in developed countries, the major source of salt in the Chinese diet is salt added by the consumers themselves during food preparation.[15,16] In response to the high salt intake, Action on Salt China (ASC) unit was set up in 2017 with the purpose of reducing salt intake by implementing a comprehensive national salt reduction programme.[17] ASC is funded by the National Institute for Health Research (NIHR) of UK, led by Queen Mary University of London, The George Institute China, Chinese Center for Disease Control and Prevention (China CDC), and several other key agencies in China. ASC team have designed, a series of programmes including health education campaigns and salt reduction in pre-packaged food as well as four clusters randomized controlled trials (RCT) to develop and test specific intervention packages

For peer review only

targeting different settings or populations. The four RCTs are (1) Application-based Intervention Study (AIS) targeting schoolchildren and their families;[18] (2) Housewife-based Intervention study (HIS) supporting using less salt in home cooking; (3) Restaurant-based Intervention Study (RIS), targeting salt intake from restaurants; and (4) Comprehensive Intervention Study (CIS) as a scale-up study, i.e. the study which is reported in this paper.

CIS was designed to simulate real world scale up, in which all the available interventions adopted by other RCTs will be provided to the local government of participating counties. The aim is to evaluate the acceptability of each components of intervention, the effectiveness and cost-effectiveness of the intervention after the one-year intervention, the long-lasting effectiveness after the intervention stopped for one year, and finally to provide evidence for national scale-up. This paper describes the study design, implementation and current status of CIS.

METHODS AND ANALYSIS

Study Setting and Overall Design

Considering multiple aspects such as geographical location, economic level and dietary habits, this project will be carried out in six provinces including Hebei, Heilongjiang, Jiangxi, Hunan, Sichuan and Qinghai, covering north and south, east and west, and central China.

CIS is designed as a cluster RCT, launched in September 2018 and to be completed by the end of 2020. The clusters are 48 towns (called "streets" in urban areas, but only "town" is used hereafter for simplification) selected from 12 counties (named district in urban areas, but only "county" is used hereafter) across the 6 provinces. Two counties are selected from each province, mainly from rural or suburb areas where people live in a relatively isolated local environment unlike those in central urban area. This may help to minimize contamination among counties and towns. In each county, 4 towns, with similar population and economic development level, and not adjacent to each other, are selected with the purpose of avoiding imbalance on potential confounders between

For peer review only

intervention and control groups due to the small number (48) of clusters and contamination of intervention to control group. 56 eligible participants are selected from each town to receive randomized treatment, intervention or not, and evaluation during a two years follow-up. The 4 towns are evenly randomized into the intervention or control group after the baseline survey has been completed for all the four towns within a county. The randomization methodology is acknowledged in advance to all the investigators at provincial, county and township levels, but the result of randomization is concealed until the centralized randomization finished.

The implementation of intervention is led by county investigators with the support from local governments, but the delivery of the intervention package is conducted at the town level. To minimize contamination, all the intervention activities are required to be conducted within the intervention town. The intervention will be carried out for the first 12 months. The effectiveness of intervention will be evaluated after completion of the 12 months' intervention (mid-term assessment) and after another 12 months' follow-up to examine its long-lasting effectiveness (endpoint assessment).

Study Population and participant recruitment

The target population is all adult residents in study sites. In this study, the inclusion criteria for participants invited for evaluation are (1) age: 18-75 years; (2) maximum of one family member per family; (3) local residents for over 6 months, with no plan to move within 24 months; (4) agreement to sign informed consent form. The exclusion criteria are (1) pregnant women and those in lactation period; (2) individuals who currently participates in any other clinical trials; (3) those with severe psychiatric or physical diseases that might impact intervention and follow-up; (4) individuals who are unable or not suitable to collect 24-hour urine due to the following conditions: a) aconuresis; b) acute/chronic urinary tract infection, vaginal infection and perianal infection; c) acute hemorrhagic diseases in urinary tract, vagina and digestive tract; d) severe vomiting and diarrheic symptoms.

A two-stage sampling is conducted to recruit participants. Firstly, two villages (named

For peer review only

committees in urban areas) are randomly selected, and then 28 eligible participants are randomly selected from each village, i.e. 56 participants for each town. The procedure of village and participant selection is conducted by county investigators with the support of a specially designed smartphone application. To fulfil the random selection, the names of villages as well as the names of residents in the selected villages need to be uploaded to the server through the app, and a centralized randomization result will be presented through the app to the county investigators. The reasons for excluding individuals are also recorded through the app.

Intervention

In order to promote all intervention components for salt reduction, a multi-section engagement strategy is recommended to local governments at county, township and village levels. The government agencies and other major stakeholders to be engaged also include local centers for disease control and prevention (CDC), women federations, publicity department, hospitals, schools, restaurants, supermarkets, etc.. The county CDCs will lead the intervention at township level, including mass media publicity and education, interventions by communities, schools and catering units, and salt reduction interventions based on primary care institutions. Potential contamination may exist because the intervention is led by investigators at county level and residents in control group may visit people or eat at restaurants in intervention towns. Not adjacent to each other and restricting intervention within intervention towns will minimize the contaminations. The overall major interventions targeting different populations or settings within the intervention towns are summarized below.

Salt reduction publicity within the intervention towns Each county mobilizes the whole township society in intervention group by carrying out various theme publicity activities for salt reduction. These include (1) to carry out health knowledge promotion by training and distributing brochures and disseminate core information on salt reduction leveraging publicity days or important holidays at least twice a year, such as *World Salt Reduction Week*, *National Nutrition Week*, etc.; (2) at least once a year to organize a mass cultural and publicity activities on "Salt and Health", using local

For peer review only

popular forms, such as knowledge contest, family health gastronomy cooking contest or other activities, to create a strong action atmosphere to reduce salt; (3) to establish a good public salt reduction environment such as healthy park, healthy road, healthy edible oil station, etc.; and (4) to promote salt and health knowledge and skills through posters and self-media such as WeChat public account to increase the coverage of audiences, especially young and middle-aged groups.

In the process of mass publicity, we encourage to leverage the local culture and customs, use innovative forms and content, and encourage that the form is diverse, the coverage is wide, and the content is updated in a timely manner. At the same time, contamination to the control towns must be minimized by limiting the activities within the intervention towns.

Salt reduction based on community All communities/villages in the intervention towns try to establish salt reduction environment by putting up posters, hanging slogans and distributing pamphlets in conspicuous places. Organizing community or village family chefs and family members to conduct at least one training on salt reduction knowledge and skills every year, or carry out other forms of community themed activities, and distribute salt limiting spoons and other intervention tools. The salt restriction spoon is a plastic spoon specially designed to hold salt during cooking. The salt per spoon is 2g, which is convenient for home cooks to count and control the salt used during cooking. For the community/village family chefs and family members, the family salt intake monitoring activities carry out through the "Health Salt" WeChat applet or the salt intake evaluation booklet. Eight standards, no more than 2 minutes, audios are provided for loudspeaker broadcasting for all villages in the intervention towns.

Salt reduction based on school In all the schools in the intervention town, publicity posters should be put up in the publicity boards or school canteens. And the school recess broadcast can be used to publicize the knowledge of salt reduction, so as to create a good campus environment for salt reduction. Salt and health training activities are conducted at least once a year, using opportunities such as centralized teacher training or school parent-teacher meetings. Or in combination with local conditions, carry out

For peer review only

"AppSalt use activities" (AppSalt is an app-based platform designated for salt reduction through primary school, which is the key intervention in AIS[18]) "previous section of salt reduction health education class", "watch a salt reduction science animation", "salt reduction handwritten newspaper" and other forms of school salt reduction publicity activities. In addition, we also need to actively promote the use of AppSalt by students' parents in combination with local conditions, by means of training or school WeChat group, or organize a class to carry out the use of AppSalt.

Salt reduction based on restaurant Salt reduction activities will be carried out in restaurants, and canteens at workplaces located in the intervention towns. The knowledge of salt reduction will be publicized through posters, table decorations, and accessible brochures, so as to create a restaurant environment conducive to salt reduction. In the restaurants and the canteens, the catering chiefs are provided with standardized training at least one a year on how to reduce the amount of salt and salty sauces used during cooking at least once a year. Consumers are encouraged to order food with less salt by waiters/waitresses.

Salt reduction based on primary care service This project also covers the community health service under the jurisdiction of town (township hospitals) and village (village clinics) by publicizing salt reduction knowledge in the form of posters, accessible brochures or banners in the hospitals and clinics. If available, these facilities can broadcast salt reduction publicity videos. The county CDCs will provide training at least twice a year for all the care providers under the jurisdiction of the intervention towns, by combining with the routine training of national basic public health service. All primary health care institutions should hold lectures and guidance on salt reduction at least twice a year. Primary care providers should deliver salt reduction knowledge and skills during the routine outpatient clinics to promote salt reduction and prevention of hypertension for the visiting patients.

Theoretically, as mentioned above, although some salt reduction activities are mandatory, it is acceptable for the local governments to select some of the intervention tools or materials and add some if they think reasonable. But the degree, coverage and

For peer review only

cost must be recorded during the project implementation. No additional intervention on salt reduction will be conducted among the control group in Year 1. In Year 2, we will try to equally deliver a national scale-up through the national CDC system, with no differentiate input or support among different areas, , including those originally randomized into the control group. The hypotheses is that the national scale-up has the same impact to the two groups, and the difference on 24-hour urinary sodium excretion at the end of year 2 can reflex the pure long-lasting effectiveness of the intervention conducted in year 1.

Sample size

Our recently completed Shandong salt reduction project with similar comprehensive intervention showed that the before-after effectiveness of sodium reduction was 37 mmol/d[19]. Considering the before-after design may overestimate the effectiveness of intervention, we expect that our study will reduce sodium intake by at least 25 mmol/d (1.46 g/d salt) from baseline with comparison to the control group. The target sample size will have 80% power to detect a change of 25mmol/d. According to the research design, we randomly select 2688 eligible participants from 48 towns (56 each) in 6 provinces. Assuming the maximum drop rate is 20% for towns (from 48 to 40) and 10% for participants (from 56 to 50) within the two years follow-up, this sample size and sampling method would have 80% power to detect a difference of 25 mmol/d between the group means assuming the standard deviation is 85.0 mmol/d and intraclass correlation coefficient (ICC) is 0.080 at village level, with a two-sided analyses and a significance level of 0.05. The parameter estimations for effectiveness, standard deviation and ICC are also based on Shandong study, and also similar to the results in a cluster RCT conducted in China.[7]

Outcomes and outcome assessment

The primary outcome is salt intake measured by 24-hour urinary sodium exertion. The secondary outcomes include the change in salt-related knowledge, attitude and practice (KAP), and blood pressure.

For peer review only

All outcome assessments including questionnaires, physical measurements, and 24-hour urine collection, will be carried out at baseline, i.e. before randomization, at mid-term (i.e. after 1 year intervention), and at endpoint (i.e. two years from baseline). Both the intervention and the control towns will be assessed in exactly the same way in parallel.

The questionnaire survey will be conducted a face-to-face by trained and qualified investigators through a mobile electronic data collection system.[20] According to the content and sequence of the questionnaire, the basic information of the respondents and relevant behavioral risk factors, knowledge, attitudes and behaviors related to salt reduction and prevention and control of hypertension, hypertension and related expenses were collected. Physical measurements are accurately measured by trained researchers using calibrated measuring instruments, including height, weight, waist circumference, blood pressure, and heart rate.

During the 24-hour urine collection, we asked the respondents to empty the bladder, record the start time, dispense the urine collection equipment, and inform the respondents of the urine retention precautions, and specify the collection time for the next day. When retrieving the urine collection equipment, we need to ask for the last urination time. If the respondent does not remember the time of the last urine collection, then the final urine should be collected on site and the end time recorded. If any of the following three problems occur, it is determined that the urine sample is unacceptable: (1) forget to collect or splash urine more than 10% of the total; (2) urine is contaminated with blood, stool or other impurities; (3) excessive sweating, diarrhea or vomiting during collection. If any of the above situations is reported, another 24-hour urine collection should be re-scheduled. Finally, the qualified urine is sealed and transported to the laboratory for unified testing. The test included urinary sodium, urinary potassium, creatinine and albumin.

All local staff participating in the field investigation will be given appropriate training including tests. Only those who pass the tests can take part in the field works. And they will be compensated accordingly.

For peer review only

DATA COLLECTION AND ANALYSIS

Data collection methods

Case report forms (CRFs) will be designed to collect data of both the baseline survey and evaluation, including demographic characteristics, salt-related KAP, blood pressure, and 24-hour urinary sodium levels. Besides such data collected at the baseline, participants lost and reasons for loss need to be recorded both at the stages of 12-month follow-up and longer-lasting effectiveness evaluation at 24 months. Electronic data capture technology will be employed to support data collection.

Data management

The data of this survey was collected through the mobile electronic data acquisition system (mEDC),[20] which was developed by Beijing University of Aeronautics and Astronautics. Relevant data such as questionnaires, physical measurements, urine collection and intervention processes were collected by the mEDC system.

Statistical Analysis

The effectiveness of the intervention package on primary and secondary outcomes will be evaluated at 12 months after intervention, and at 24 months after the intervention stopped for one year. The linear mixed models will be used to model outcome measure at 12 months (primary analysis), with adjustment for the baseline variable same as the analysed outcome, and with participants nested within village units and villages nested within towns. The group difference will be estimated using least squares estimation. To account for missing data on continuous outcomes, we will use the likelihood based random effects model that uses all available data to provide valid estimates of the intervention effects when data are missing at random. If the missing data is above 5%, various missing value imputation methods will be adopted as sensitivity analyses to examine the robustness of the conclusions of the primary analysis.

When evaluating the longer-lasting effectiveness of the intervention package at 24

For peer review only

months, the same methods as mentioned above will be applied, with replacing the major/secondary outcomes at 12 months by those at 24 months. We assume the effect of the scale-up will be offset between the intervention and control group.

SAS will be used for the analyses. Results will be reported as mean, SD, SE, and 95% confidence interval when appropriate. All analyses are two sided, and $P<0.05$ is considered significant.

Process monitoring and evaluation

In order to ensure to supervise the fidelity and adaption to the intervention, process monitoring will be carried out throughout the intervention period. Process monitoring includes evaluation of indicator system and method. For each county, governance, working system, and effective intervention plan must be ready before the initiation of intervention. During the implementation, all the intervention will be recorded and reported on a quarterly basis according to the activity plan. The report includes the content of the activity, the time of the event, the implementer, the participants, and other relevant documents, photos and objects and so on. The national and provincial supervision teams will visit the intervention sites quarterly to check the construction of the salt reduction environment, check the posting and placement of posters, brochures and other promotional materials, and understand the distribution of materials, lectures and training.

At the end of year 1 and year 2, a systematic semi-structured interviews will be conducted separately to evaluate the fidelity and acceptability of each components of intervention. We will adopt an approach consistent with the UK MRC Guidelines for process evaluations of complex intervention[21]. This will enable us to answer whether the intervention is effective, and what are the barriers and enablers for the potential scale-up. A combination of in depth interviews will be conducted with study participants, staff who delivered the intervention at county, town and village level, and policy makers, as well as interrogation of data collected during the trial.

Economic evaluation

For peer review only

Economic evaluations will be carried out from health sector perspective to compare the comprehensive intervention with usual care, and it will entail two components: a trial-based economic evaluation and a modelled economic evaluation of long-term costs and outcomes. Intervention costs will include those in delivering the intervention which shall be recorded by the county investigators, but exclude any research and development costs. The trial-based economic evaluation will be assessed initially in terms of incremental cost per unit reduction in salt intake and systolic BP. The modelled economic evaluation with discounting will examine the cost, survival, quality of life over lifetime, via capturing various health states (including death and CVD events) to estimate incremental cost per life year saved and cost per Quality-Adjusted Life Year gained. The transition probabilities across health states and costs attached to various health states will be based on literature and the long-term effects of the reduction in salt intake or systolic BP will be derived from the trial findings and/or literature of disease progression. Sensitivity analyses will be carried out to estimate uncertainty about the primary findings associated with varying key parameters.

Project status and timelines

Preparations are made from April 2017 to August 2018. The baseline assessment was initiated from September to December 2018. 2688 eligible participants have been successfully recruited from 48 towns (4 of them located in urban areas) of 12 counties (2 of them located in urban areas) and completed the baseline survey. Since two rounds of effect evaluations are to be carried out after 12 months and 24 months, a mid-term evaluation will be conducted by the end of 2019, and the final follow-up evaluation will be conducted in December 2019.

Patient and Public Involvement

According to the actual situation in the locality, the investigation site adopts various forms to carry out propaganda and mobilization work, and introduces the meaning and purpose of the investigation to the residents. Rely on the leadership and support of the local government and grassroots organizations, master the situation, make appointments, and strive to understand, support and cooperate with the respondents.

For peer review only

After determining the identity of the investigator, we will sign an informed consent form with the investigator, then conduct a questionnaire survey, body measurements, and urine collection.

ETHICS AND DISSEMINATION

Research Ethics Approval and Consent

The study has been reviewed and approved by the Institutional Review Board of the National Center for Chronic and Noncommunicable Disease Control and Prevention, the Chinese Center for Disease Control and Prevention (201807), and Queen Mary Research Ethics Committee. Written consent will be obtained from all participants who will be free to discontinue their participation at any time, with no explanation required.

Consent or Assent

Consent for participation in the project will be sought both at the cluster level and the individual level. Cluster level consent of the community will be obtained through a consultation process involving government (at provincial, county and town levels) and village leaders. The project, including the process of random assignment of communities to intervention and control conditions and the nature of interventions, will be explained at a face-to-face meeting. Questions will be answered and all relevant stakeholder groups invited to consult with their members and reflect upon the project. Individual consent for participation in outcome surveys will be obtained from all persons selected in a standard manner via provision of a participant information sheet, explanation and discussion as required, and the collection of written consent from those willing to take part.

Written consent will be obtained from all participants who will be free to discontinue their participation at any time, with no explanation required.

Acknowledgements

The author would like to thank street/community residents, catering companies, school

For peer review only

teachers, parents of primary school students, primary care staff, and village, town and county leaders for their opinions on the development of the intervention program.

Contributors

PZ and JW conceived the project. JX, ML, YB, YL, JW, FJH, GAM and PZ participated in the design and implementation of the project. JX, BT, ML, YB, WY, XZ, ZX, JH, DJ, JS, YL, JW, FJH, GAM and PZ facilitates Patient and Public Involvement and were responsible for setting up the study in each site. BT and JX wrote the first draft of the manuscript, they contributed equally to this paper. All authors contributed to the refinement of the study protocol and approved the final manuscript.

Funding

This work is supported by the National Institute of Health Research (NIHR) (NIHR Global Health Research Unit Action on Salt China at Queen Mary University of London) using Official Development Assistance (ODA) funding (16/136/77). The views expressed in this publication are those of the author(s) and not necessarily those of the NIHR or the Department of Health and Social Care.

Competing interests

FJH is a member of the Consensus Action on Salt & Health (CASH) group, a non-profit charitable organisation, and its international branch World Action on Salt & Health (WASH) and does not receive any financial support from CASH or WASH. GAM is the Chairman of Blood Pressure UK (BPUK), Chairman of CASH and Chairman of WASH and does not receive any financial support from any of these organisations. BPUK, CASH and WASH are non-profit charitable organisations. All other authors have no competing interest to declare.

Dissemination policy

The findings of this study will be disseminated through discussion or presentations at selected conferences, peer-reviewed publications and the general media.

For peer review only

REFERENCES

1 World Health Organization. Reducing risks, promoting healthy life. Geneva, Switzerland: World Health Organisation, 2002. http://www.who.int/whr/2002 (Accessed 13 Jul 2019).

2 Intersalt Cooperative Research Group. Intersalt: an international study of electrolyte excretion and blood pressure. Results for 24hour urinary sodium and potassium excretion. *BMJ* 1988, 297:319-328.

3 He FJ, MacGregor GA. Reducing population salt intake worldwide: from evidence to implementation. *Prog Cardiovasc Dis* 2010, 52:363-382.

4 He J, Gu D, Chen J, et al. Premature deaths attributable to blood pressure in China: a prospective cohort study. *Lancet* 2009, 374:1765-1772.

5 Wu Y, Huxiey R, Li L, et al. Prevalence, awareness, treatment, and control of hypertension in China: data from the China National Nutrition and Health Survey 2002. *Circulation* 2008, 118:2679-2686.

6 Zhou BF, Stamler J, Dennis B, et al.Nutrient intakes of middle-aged men and women in China, Japan, United Kingdom, and United States in the late 1990s:the INTERMAP study. *J Hum Hypertens* 2003, 17:623-630.

7 He FJ, Wu Y, Feng XX, et al. School based education programme to reduce salt intake in children and their families (School-EduSalt): cluster randomised controlled trial. *BMJ* 2015, 350:h770

8 He FJ, Li J, Macgregor GA. Effect of longer term modest salt reduction on blood pressure: cochrane systematic review and meta-analysis of randomised trials. *BMJ* 2013, 346: f1325.

9 Aburto NJ, Ziolkovska A, Hooper L, et al. Effect of lower sodium intake on health: systematic review and meta-analyses. *BMJ* 2013, 346: f1326.

10 He FJ, MacGregor GA. Salt reduction lowers cardiovascular risk: meta-analysis of outcome trials. *Lancet* 2011, 378:380-382.

11 He FJ, Campbell NRC, Ma Y, MacGregor GA, et al. Errors in estimating usual sodium intake by the Kawasaki formula alter its relationship with mortality: implications for public health. *Int J Epidemiol* 2018, 47(6):1784-1795.

12 Bibbins-Domingo K, Chertow GM, Coxson PG, et al. Projected effect of dietary salt reductions on future cardiovascular disease. *N Engl J Med* 2010, 362:590-599.

13 National Institute for Health and Clinical Excellence(NICE). Guidance on the prevention of cardiovascular disease at the population level. http://guidance.nice.org.uk/PH25 (Accessed 13 Jul 2019).

14 World Health Organization. First global ministerial conference on healthy lifestyles and noncommunicable disease control. https://www.who.int/nmh/events/moscow_ncds_2011/en/ (Accessed 13 Jul 2019).

15 Anderson CA, Appel LJ, Okuda N, et al. Dietary sources of sodium in China, Japan, the United Kingdom, and the Untied States, women and men aged 49 to 59 years: the INTERMAP study. *J AM Diet Assoc* 2010, 110:736-745.

For peer review only

16 Xu J, Wang M, Chen Y, et al. Estimation of salt intake by 24-hour urinary sodium excretion: a cross-sectional study in Yantai, China. *BMC Public Health* 2014, 14:136.

17 He FJ, Zhang PH, Li Y, et al. Action on Salt China. *Lancet* 2018,392(10141):7-9.

18 He FJ, Zhang PH, Luo R, et al. An Application-based programme to reinforce and maintain lower salt intake (AppSalt) in schoolchildren and their families in China. *BMJ Open* 2019,9(7): e027793.

19 Chen X, Guo X, Ma J, et al. Urinary sodium or potassium excretion and blood pressure in adults of Shandong province, China: preliminary results of the SMASH project. *J Am Soc Hypertens* 2015, 9(10): 754-762.

20 Zhang J, Sun L, Liu Y, et al. Mobile Device-Based Electronic Data Capture System Used in a Clinical Randomized Controlled Trial: Advantages and Challenges. *J Med Internet Res* 2017, 19(3):e66.

21 Moore GF, Audrey S, Barker M, et al. Process evaluation of complex interventions:

Medical Research Council guidance. BMJ, 2015, 350:h1258.

For peer review only

BMJ Open

A town level comprehensive intervention study (CIS) to reduce salt intake in China: Protocol for a cluster randomized controlled trial

Journal:	*BMJ Open*
Manuscript ID	bmjopen-2019-032976.R3
Article Type:	Protocol
Date Submitted by the Author:	13-Dec-2019
Complete List of Authors:	Xu, Jianwei; National Center for Chronic and Noncommunicable Disease Control and Prevention, Chinese Center for Disease Control and Prevention,<br>Tang, Biwei; National Center for Chronic and Noncommunicable Disease Control and Prevention, Chinese Center for Disease Control and Prevention; Inner Mongolia Medical University<br>Liu, Min; National Center for Chronic and Noncommunicable Disease Control and Prevention, Chinese Center for Disease Control and Prevention<br>Bai, Yamin; National Center for Chronic and Noncommunicable Disease Control and Prevention, Chinese Center for Disease Control and Prevention<br>Yan, Wei; Jiangxi Provincial Center for Disease Control and Prevention<br>Zhou, Xue; Heilongjiang Provincial Center for Disease Control and Prevention<br>Xu, Zhihua; Qinghai Provincial Center for Disease Control and Prevention<br>He, Jun; Sichuan Provincial Center for Disease Control and Prevention<br>Jin, Donghui; Hunan Provincial Center for Disease Control and Prevention<br>Sun, Jixin; Hebei Provincial Center for Disease Control and Prevention<br>Li, Yuan; The George Institute for Global Health, Peking University Health Science Centre<br>He, Feng; Queen Mary University of London, Wolfson Institute of Preventive Medicine<br>MacGregor, Graham; Queen Mary University of London, Wolfson Institute of Preventive Medicine<br>Wu, Jing; National Center for Chronic and Noncommunicable Disease Control and Prevention, Chinese Center for Disease Control and Prevention<br>Zhang, Puhong ; The George Institute at Peking University Health Science Center, Diabetes Program
**Primary Subject Heading**:	Epidemiology
Secondary Subject Heading:	Epidemiology, Public health
Keywords:	Hypertension < CARDIOLOGY, EPIDEMIOLOGY, STATISTICS & RESEARCH METHODS, PUBLIC HEALTH

SCHOLARONE™
Manuscripts

I, the Submitting Author has the right to grant and does grant on behalf of all authors of the Work (as defined in the below author licence), an exclusive licence and/or a non-exclusive licence for contributions from authors who are: i) UK Crown employees; ii) where BMJ has agreed a CC-BY licence shall apply, and/or iii) in accordance with the terms applicable for US Federal Government officers or employees acting as part of their official duties; on a worldwide, perpetual, irrevocable, royalty-free basis to BMJ Publishing Group Ltd ("BMJ") its licensees and where the relevant Journal is co-owned by BMJ to the co-owners of the Journal, to publish the Work in this journal and any other BMJ products and to exploit all rights, as set out in our licence.

The Submitting Author accepts and understands that any supply made under these terms is made by BMJ to the Submitting Author unless you are acting as an employee on behalf of your employer or a postgraduate student of an affiliated institution which is paying any applicable article publishing charge ("APC") for Open Access articles. Where the Submitting Author wishes to make the Work available on an Open Access basis (and intends to pay the relevant APC), the terms of reuse of such Open Access shall be governed by a Creative Commons licence – details of these licences and which Creative Commons licence will apply to this Work are set out in our licence referred to above.

Other than as permitted in any relevant BMJ Author's Self Archiving Policies, I confirm this Work has not been accepted for publication elsewhere, is not being considered for publication elsewhere and does not duplicate material already published. I confirm all authors consent to publication of this Work and authorise the granting of this licence.

For peer review only

A town level comprehensive intervention study (CIS) to reduce salt intake in China: Protocol for a cluster randomized controlled trial

Jianwei Xu[1], Biwei Tang[1,2], Min Liu[1], Yamin Bai[1], Wei Yan[3], Xue Zhou[4], Zhihua Xu[5], Jun He[6], Donghui Jin[7], Jixin Sun[8], Yuan Li[9,10], Feng J He[11], Graham A MacGregor[11], Jing Wu[1], Puhong Zhang[9,10]

[1]National Center for Chronic and Noncommunicable Disease Control and Prevention, Chinese Center for Disease Control and Prevention, Beijing, China

[2]Inner Mongolia Medical University, Hohhot, China

[3]Jiangxi Provincial Center for Disease Control and Prevention, Nanchang, China

[4]Heilongjiang Provincial Center for Disease Control and Prevention, Harbin, China

[5]Qinghai Provincial Center for Disease Control and Prevention, Xining, China

[6]Sichuan Provincial Center for Disease Control and Prevention, Chengdu, China

[7]Hunan Provincial Center for Disease Control and Prevention, Changsha, China

[8]Hebei Provincial Center for Disease Control and Prevention, Shijiazhuang, China

[9]The George Institute for Global Health at Peking University Health Science Center, China.

[10]Faculty of Medicine, University of New South Wales, Australia.

[11]Wolfson Institute of Preventive Medicine, Barts and The London School of Medicine & Dentistry, Queen Mary University of London, UK

Correspondence to:

Jing Wu

National Center for Chronic and Noncommunicable Disease Control and Prevention, Chinese Center for Disease Control and Prevention

For peer review only

No. 27, Nanwei Road

Xicheng District | Beijing, 100050 P.R. China

Tel: +86 10 8315 4663

E-mail: wujingcdc@163.com

Puhong Zhang

The George Institute for Global Health at Peking University Health Science Center,

Level 18, Tower B, Horizon Tower, No. 6 Zhichun Rd,

Haidian District | Beijing, 100088 P.R. China

Tel: +86 10 8280 0577 ext 512

Fax: +8610 8280 0177

E-mail: zpuhong@georgeinstitute.org.cn

A total of 5106 words, excluding title page, abstract, references, figures and tables.

For peer review only

ABSTRACT

Introduction: Salt intake in China (≈12g/d) is more than twice the upper limit recommended by the World Health Organization(5g/d). To reduce salt intake, Action on Salt China (ASC) was launched in 2017. As one of four randomized controlled trials (RCTs) in the ASC programme, a comprehensive intervention study (CIS) was designed to test whether all the components of the interventions adopted by other RCTs are acceptable, scalable and effective when provided to a region in the real world.

Methods and analysis: Using a cluster RCT design, 2688 participants were selected from 48 towns (clusters) in 12 counties in 6 provinces and assigned to the intervention group or the control group. Randomization was performed after the baseline survey was completed. Information on salt-related knowledge, attitude and practice (KAP), blood pressure and 24-hour urinary sodium were collected. The intervention includes government engagement, health education and other intervention components targeting restaurants, home cooks, and primary school students and their families that have been used in other RCTs. The control group will not receive the intervention. The project will be followed up for two years, with the intervention being carried out for the first year only. The primary outcome is salt intake measured by 24-hour urinary sodium excretion after one year. The secondary outcomes are the long-lasting effectiveness on salt intake and blood pressure measured by the same method, as well as salt-related KAP and blood pressure at the one- and two-year follow-ups. Process evaluation and health economics analysis will be conducted as well.

Ethics and dissemination: The study was reviewed and approved by the Institutional Review Board of the National Center for Chronic and Noncommunicable Disease Control and Prevention, the Chinese Center for Disease Control and Prevention, and Queen Mary Research Ethics Committee. Results will be disseminated through presentations, publications and social media.

Trial registration number: ChiCTR1800018119

Key words: Hypertension; salt; cluster randomized control trial; intervention

For peer review only

Strengths and limitations of this study

1. This study is the first randomized controlled trial of comprehensive intervention in urban salt reduction in China.

2. Our comprehensive intervention measures cover different groups of people and different places, and benefit a wide range of people.

3. The shortcoming of this study is that the implementation of comprehensive salt reduction interventions requires the strong support and cooperation of local governments and institutions.

For peer review only

INTRODUCTION

Studies have shown that high salt (sodium) intake is one of the causes of high blood pressure levels and hypertension and that hypertension is an independent risk factor for stroke and coronary heart disease, as it accounts for 62% of strokes and 49% of coronary heart disease cases.[1-3] In China, there were approximately 177 million people with hypertension in 2002, and high blood pressure (BP) attributed to 2.33 million cardiovascular disease (CVD) deaths in 2005.[4] Hypertension is a 'silent killer', and 75% of hypertensive Chinese individuals are not aware that they have high BP.[5] In China, salt intake is very high, with an average intake rate of 12-14 g/day.[6,7] There is compelling evidence in adults that a modest reduction in salt intake lowers BP and reduces cardiovascular risk.[3,8-11] Salt reduction is one of the most cost-effective ways to prevent CVD in high-income as well as low-income and middle-income countries.[12,13] The daily salt intake that is currently recommended by the World Health Organization (WHO) is 5 g (the Chinese government recommends 5 g). According to the results of the 2002 National Nutrition Survey, Chinese adults' daily salt intake is twice that of China's recommended dietary guidelines. The World Health Organization has recommended salt reduction as one of the top three prioritized actions to tackle the global non-communicable disease crisis.[14]

Unlike in diets in developed countries, the major source of salt in the Chinese diet is salt added by the consumers themselves during food preparation.[15,16] In response to the high salt intake, the Action on Salt China (ASC) group was established in 2017 to reduce salt intake by implementing a comprehensive national salt reduction programme.[17] ASC is funded by the National Institute for Health Research (NIHR) of the UK and led by the Queen Mary University of London, The George Institute China, Chinese Center for Disease Control and Prevention (China CDC), and several other key agencies in China. The ASC team has designed a series of programmes, including campaigns for health education and salt reduction in pre-packaged food, as well as four cluster randomized controlled trials (RCTs) to develop and test specific interventions targeting different settings or populations. The four RCTs include the

For peer review only

(1) application-based intervention study (AIS) targeting schoolchildren and their families;[18] (2) housewife-based intervention study (HIS) supporting the use of less salt in home cooking; (3) restaurant-based intervention study (RIS), targeting salt intake from restaurants; and (4) comprehensive intervention study (CIS), which is a scale-up study, i.e., the study that is reported in this paper.

The CIS was designed to simulate the real world on a large scale, and all the available interventions adopted in the other RCTs will be provided to the local governments of the participating counties. The aims are to evaluate the acceptability of each component of the intervention, the effectiveness and cost-effectiveness of one year of the intervention, and the long-lasting effectiveness of the intervention one year after it is terminated and to provide evidence for a national scale-up. This paper describes the study design, implementation and current status of the CIS.

METHODS AND ANALYSIS

Study Setting and Overall Design

Considering multiple aspects, such as the geographical location, economic level and dietary habits, this project will be carried out in six provinces, including Hebei, Heilongjiang, Jiangxi, Hunan, Sichuan and Qinghai, which cover the north, south, east, west, and central regions in China.

The CIS was designed as a cluster RCT; it was launched in September 2018 and is expected to be completed by the end of 2020. The clusters are 48 towns (called "streets" in urban areas, but only "town" is used hereafter for simplification) selected from 12 counties (named "districts" in urban areas, but only "county" is used hereafter) across the 6 provinces. Two counties are selected from each province, mainly from rural or suburban areas where people live in a relatively isolated local environment, unlike those in central urban areas. This process may help to minimize contamination among counties and towns. In each county, 4 towns that have similar population and economic development levels and are not adjacent to each other are selected to prevent imbalances in potential confounders between the intervention and control groups that

For peer review only

may occur due to the small number (48) of clusters and contamination of intervention to the control group. Fifty-six eligible participants were selected from each town and were randomly allocated to receive either the intervention or no intervention and an evaluation during a two-year follow-up period. The 4 towns are evenly and randomly allocated into the intervention or control group after the baseline survey has been completed for all four towns within a county. The randomization methodology is shared in advance with all the investigators at provincial, county and township levels, but the randomization results are concealed until the centralized randomization process is complete.

The implementation of the intervention is led by county investigators with support from local governments, but the intervention is conducted at the town level. To minimize contamination, all the intervention activities must be conducted within the towns participating in the intervention. The intervention will be carried out for the first 12 months. The effectiveness of the intervention will be evaluated after the completion of the 12-months intervention (mid-term assessment) and after another 12 months following the completion of the intervention to examine its long-lasting effectiveness (endpoint assessment).

Study Population and participant recruitment

The target population includes all adult residents in the study sites. In this study, the inclusion criteria for the participants invited for evaluation are those (1) aged 18-75 years; (2) who do not have another family member participating in the study (a maximum of one family member per family is included); (3) who were considered local residents for over 6 months and have no plans to move within the next 24 months; and (4) who agreed to sign an informed consent form. The exclusion criteria are (1) pregnant women and those in the lactation period; (2) individuals who currently participate in any other clinical trials; (3) those with severe psychiatric or physical diseases that might impact the intervention and follow-up; and (4) individuals who are unable or for whom it is not suitable to collect 24-hour urine due to the following conditions: a) aconuresis; b) acute/chronic urinary tract infection, vaginal infection and

For peer review only

perianal infection; c) acute haemorrhagic diseases in the urinary tract, vagina and digestive tract; or d) severe vomiting and diarrheic symptoms.

Two-stage sampling is conducted to recruit participants. First, two villages (named committees in urban areas) are randomly selected, and then, 28 eligible participants, i.e., 56 participants for each town, are randomly selected from each village. The village and participant selection procedure is conducted by county investigators with a specially designed smartphone application. For the random selection procedure, the names of the villages as well as the names of the residents in the selected villages need to be uploaded to the server through the app, and a centralized randomization result will be presented through the app to the county investigators. The reasons for excluding individuals are also recorded through the app.

Intervention

To promote all intervention components for salt reduction, a multi-section engagement strategy is recommended to local governments at the county, township and village levels. The government agencies and other major stakeholders to be engaged also include local centres for disease control and prevention (CDCs), women federations, publicity departments, hospitals, schools, restaurants, supermarkets, etc. The county CDCs will lead the intervention at the township level, including mass media publicity and education efforts, interventions for communities, schools and catering units, and salt reduction interventions based in primary care institutions. Potential contamination may exist because the intervention is led by investigators at the county level, and residents in the control group may visit people or eat at restaurants in towns participating in the intervention. Selecting towns that are not adjacent to each other and restricting the intervention to the towns participating in the intervention will minimize the amount of contamination. The overall major interventions targeting different populations or settings within the towns participating in the intervention are summarized below.

Salt reduction publicity within the towns participating in the intervention Each county

For peer review only

will mobilize the whole township society in the intervention group by carrying out various themed publicity activities for salt reduction. These include (1) promoting health knowledge by training and distributing brochures and disseminating core information on salt reduction and by leveraging at least two publicity days or important holidays each year, such as *World Salt Reduction Week* and *National Nutrition Week*; (2) organizing mass cultural and publicity activities on "Salt and Health" at least once a year using local popular forms, such as knowledge contests, family health gastronomy cooking contests or other activities, to create an atmosphere dedicated to the reduction of salt intake; (3) establishing good public environments focused on the reduction of salt intake, such as healthy parks, healthy roads, and healthy edible oil stations and (4) promoting salt and health knowledge and skills through posters and social media, such as WeChat public accounts, to broaden the audiences to especially include young and middle-aged groups.

For mass publicity, we encourage leveraging the local culture and customs and using innovative forms and content, and we suggest that the form is diverse, the coverage is broad, and the content is updated in a timely manner. Moreover, contamination to the towns in the control group must be minimized by limiting the activities to only the towns participating in the intervention.

Salt reduction based on community All communities/villages in the towns participating in the intervention will try to establish a salt reduction environment by hanging posters, introducing slogans and distributing pamphlets in conspicuous places. Community or village family chefs and family members will organize at least one training event on salt reduction every year to improve the knowledge and skills of the attendants or carry out other forms of community themed activities and distribute salt-restricting spoons and other intervention tools. The salt-restricting spoon is a plastic spoon specially designed to measure salt during cooking. Each spoon holds 2 g of salt, which is convenient for home cooks to count and control the salt used during cooking. For the community/village family chefs and family members, the family salt intake monitoring activities are carried out through the “Health Salt” WeChat applet or the

For peer review only

salt intake evaluation booklet. Audio recording of eight standards, each lasting no more than 2 minutes, are provided for loudspeaker broadcasting for all villages in the towns participating in the intervention.

Salt reduction based on school In all the schools in the towns participating in the intervention, publicity posters should be put up on bulletin boards or school canteens. Information on salt reduction can be broadcast at the school during recess to create a good campus environment focused on salt reduction. Salt and health training activities are conducted at least once a year using opportunities such as centralized teacher training or school parent-teacher meetings. In combination with the local efforts, "AppSalt activities" (AppSalt is an app-based platform that was designed for promoting salt reduction in primary schools, which is the key intervention in the AIS[18]), " salt reduction-focused sessions in health education classes", "showings of salt reduction science animations", "the production of salt reduction-related handwritten newspapers" and other forms of salt reduction public activities are conducted in schools. In addition, we also need to actively encourage students' parents to use AppSalt by conducting training sessions, organizing school WeChat groups, or organizing a class on the use of AppSalt.

Salt reduction based on restaurant Salt reduction activities will be carried out in restaurants and canteens at workplaces located in the towns participating in the intervention. Information on salt reduction will be shared through posters, table displays, and brochures made publicly available to create a restaurant environment conducive to the reduction of salt. In the restaurants and the canteens, the catering chiefs are offered standardized training for at least one year on how to reduce the amount of salt and salty sauces used during cooking at least once a year. Consumers are encouraged to order food with less salt from the servers.

Salt reduction based on primary care service This project also addresses the community health services under the jurisdiction of the towns (township hospitals) and villages (village clinics) by publicizing salt reduction information in the form of posters, accessible brochures or banners in the hospitals and clinics. If possible, these facilities

For peer review only

can publicly broadcast salt reduction videos. The county CDCs will provide training at least twice a year for all the health care providers under the jurisdiction of the towns participating in the intervention by combining the training with the routine training of basic national public health services. All primary health care institutions should hold lectures and informational sessions on salt reduction at least twice a year. Primary care providers should share their salt reduction knowledge and skills during routine outpatient clinic visits to promote the reduction of salt and prevention of hypertension for the visiting patients.

Theoretically, as mentioned above, although some salt reduction activities are mandatory, it is acceptable for the local governments to select some of the intervention tools or materials and add some as they see fit. However, the degree, coverage and cost of the projects must be recorded when they are implemented. No additional interventions on salt reduction will be conducted among the control group in Year 1. In Year 2, we will try to scale the interventions evenly across the nation through the national CDC system, with no differences in the input or support among the different areas, including those originally randomized into the control group. The hypotheses are that the national scale-up has the same impact on the two groups, and the difference in 24-hour urinary sodium excretion at the end of year 2 can reflect the actual long-lasting effectiveness of the intervention conducted in Year 1.

Sample size

We recently completed Shandong salt reduction project with a similar comprehensive intervention, and it showed that the before-after effectiveness of sodium reduction was 37 mmol/d[19]. Considering that a before-after design may overestimate the effectiveness of the intervention, we expect that our intervention, compared with the control condition, will reduce sodium intake by at least 25 mmol/d (1.46 g/d salt) from baseline. The target sample size will have 80% power to detect a change of 25 mmol/d. According to the research design, we randomly selected 2688 eligible participants from 48 towns (56 each) in 6 provinces. Assuming the maximum drop rate is 20% for towns (from 48 to 40) and 10% for participants (from 56 to 50) within the two-year follow-up

For peer review only

period, this sample size and sampling method will have 80% power to detect a difference of 25 mmol/d between the group means, assuming the standard deviation is 85.0 mmol/d and intraclass correlation coefficient (ICC) is 0.080 at the village level, with a two-sided analyses and a significance level of 0.05. The parameter estimations for effectiveness, standard deviation and ICC are also based on those in the Shandong study and are similar to the results of a cluster RCT that was conducted in China.[7]

Outcomes and outcome assessment

The primary outcome is salt intake measured by 24-hour urinary sodium exertion. The secondary outcomes include the changes in salt-related knowledge, attitude and practice (KAP) and blood pressure.

All outcome assessments, including the questionnaires, physical measurements, and 24-hour urine collection, will be carried out at baseline, i.e., before randomization, at the mid-term point (i.e., after 1 year of the intervention) and at the endpoint (i.e., two years from baseline). Both the towns in the intervention group and those in the control group will be assessed in exactly the same way in parallel.

The questionnaire survey will be conducted face-to-face by trained and qualified investigators through a mobile electronic data collection system.[20] According to the content and sequence of the questionnaire, the basic information of the respondents; behavioural risk factors, knowledge, attitudes and behaviours related to the reduction of salt and prevention and control of hypertension; the presence of hypertension and related expenses will be recorded. Physical measurements, including height, weight, waist circumference, blood pressure, and heart rate, will be accurately measured by trained researchers using calibrated measuring instruments.

During the 24-hour urine collection, we will ask the respondents to empty their bladder, record the start time, dispense the urine collection equipment, inform the respondents of the urine retention precautions, and specify the collection time for the next day. When retrieving the urine collection equipment, we must ask for the last urination time. If the respondent does not remember the time of the last urine collection, then the final

For peer review only

urine sample should be collected on site, and the end time should be recorded. If any of the following three problems occur, the urine sample will be deemed unacceptable: (1) the participant forgets to collect or splashes more than 10% of the total amount of urine; (2) the urine is contaminated with blood, stool or other impurities; and (3) the participant experiences excessive sweating, diarrhoea or vomiting during collection. If any of the above situations are reported, another 24-hour urine collection should be re-scheduled. Finally, the qualified urine is sealed and transported to the laboratory for unified testing. The test included urinary sodium, urinary potassium, creatinine and albumin.

All local staff members participating in the field investigation will be given the appropriate training, which includes tests. Only the staff members who pass the tests can take part in the field work. In addition, they will be compensated accordingly.

DATA COLLECTION AND ANALYSIS

Data collection methods

Case report forms (CRFs) will be developed to collect data, including demographic characteristics, salt-related KAP, blood pressure, and 24-hour urinary sodium levels, from both the baseline survey and evaluation. In addition to the data collected at the baseline, participants lost to follow-up and the corresponding reasons must be recorded both at the 12-month follow-up and the long-lasting effectiveness evaluation at 24 months. Electronic data recording technology will be employed for the data collection.

Data management

The data from this survey were collected through a mobile electronic data acquisition system (mEDC),[20] which was developed by Beijing University of Aeronautics and Astronautics. Relevant data, such as the questionnaire results, physical measurements, and data on the urine collection and intervention processes, were collected by the mEDC system.

For peer review only

Statistical Analysis

The effectiveness of the intervention package on the primary and secondary outcomes will be evaluated at 12 months after the intervention and at 24 months after the intervention has been terminated for one year. Linear mixed models will be used to model the outcome measures at 12 months (primary analysis), with adjustments for the baseline variable, the participants nested within village units, and the villages nested within towns. The group differences will be estimated using least squares estimation. To account for missing data for the continuous outcomes, we will use the likelihood-based random effects model, which uses all available data to provide valid estimates of the intervention effects when data are missing at random. If more than 5% of the data are missing, various missing value imputation methods will be adopted as sensitivity analyses to examine the robustness of the conclusions of the primary analysis.

When evaluating the long-lasting effectiveness of the intervention package at 24 months, the methods mentioned above will be applied, and the major/secondary outcomes at 12 months will be replaced by those at 24 months. We assume that the effects of the scale-up will be similar between the intervention and control groups.

SAS will be used for the analyses. The results will be reported as the mean, SD, SE, and 95% confidence interval when appropriate. All analyses are two-sided, and $P<0.05$ is considered significant.

Process monitoring and evaluation

To assess the level of fidelity and adaption to the intervention, process monitoring will be carried out throughout the intervention period. Process monitoring includes the evaluation of the indicator systems and methods. For each county, governance, working system, and effective intervention, the plan must be ready before the initiation of the intervention. During the implementation period, all the interventions will be recorded and reported on a quarterly basis according to the activity plan. The report includes the content of the activity, the time of the event, the implementer, the participants, and other relevant documents, photos and objects. The national and provincial supervision teams

For peer review only

will visit the intervention sites quarterly to assess the development of a salt reduction environment, evaluate the posting and placement of posters, brochures and other promotional materials, and determine the distribution of materials, lectures and training.

At the end of year 1 and year 2, systematic semi-structured interviews will be conducted separately to evaluate the fidelity and acceptability of each component of the intervention. We will adopt an approach consistent with the UK MRC guidelines for the process evaluations of the complex intervention[21]. This approach will enable us to determine whether the intervention is effective and identify the barriers and enablers for the potential scale-up. A combination of in-depth interviews will be conducted with study participants, staff members who implemented the intervention at the county, town and village levels, and policy makers, and the data collected during the trial will be assessed as well.

Economic evaluation

Economic evaluations will be carried out from the health sector perspective to compare the comprehensive intervention with usual care, and the evaluations will involve two components: a trial-based economic evaluation and a modelled economic evaluation of long-term costs and outcomes. Intervention costs will include those in delivering the intervention, which shall be recorded by the county investigators, but exclude any research and development costs. The trial-based economic evaluation will be assessed initially in terms of the incremental cost per unit reduction in salt intake and systolic BP. The modelled economic evaluation with discounting will examine the cost, survival, quality of life over lifetime via capturing various health states (including death and CVD events) to estimate incremental cost per life year saved and cost per quality-adjusted life year gained. The transition probabilities across the health states and costs associated with various health states will be based on data in the literature, and the long-term effects of the reduction in salt intake or systolic BP will be identified from the trial findings and/or literature on disease progression. Sensitivity analyses will be carried out to estimate uncertainty about the primary findings associated with different key parameters.

For peer review only

Project status and timelines

Preparations were made from April 2017 to August 2018. The baseline assessment was initiated from September to December 2018. A total of 2688 eligible participants were successfully recruited from 48 towns (4 of them located in urban areas) of 12 counties (2 of them located in urban areas) and completed the baseline survey. Since two rounds of evaluations of the effects are to be carried out after 12 months and 24 months, a mid-term evaluation will be conducted by the end of 2019, and the final follow-up evaluation will be conducted in December 2019.

Patient and Public Involvement

According to the actual situation in the locality, the individuals at the investigation sites will adopt various methods of distributing propaganda and mobilization efforts and share the motivation and purpose of the investigation with the residents. They will rely on the leadership and support of the local government and grassroots organizations, lead the intervention, make appointments, and strive to understand, support and cooperate with the respondents. After the participants are selected, they will sign an informed consent form and then undergo a questionnaire survey, body measurements, and urine collection.

ETHICS AND DISSEMINATION

Research Ethics Approval and Consent

The study has been reviewed and approved by the Institutional Review Board of the National Center for Chronic and Noncommunicable Disease Control and Prevention, the Chinese Center for Disease Control and Prevention (201807), and the Queen Mary Research Ethics Committee. Written consent will be obtained from all participants, and they will be free to discontinue their participation at any time without an explanation.

Consent or Assent

Consent for participation in the project will be sought both at the cluster level and the individual level. Cluster-level consent of the community will be obtained through a

For peer review only

consultation process involving the governments (at the provincial, county and town levels) and village leaders. The project, including the process of randomly assigning communities to the intervention and control groups and the specific interventions, will be explained in a face-to-face meeting. Questions will be answered, and all relevant stakeholder groups will be invited to consult with the members of their group and reflect upon the project. Individual consent for participation in the outcome surveys will be obtained from all persons selected in a standard manner via the provision of a participant information sheet, explanation and discussion as required, and the collection of written consent from from those willing to participate.

Written consent will be obtained from all participants, and they will be free to discontinue their participation at any time without an explanation.

Acknowledgements

The author would like to thank the street/community residents, catering companies, school teachers, parents of the primary school students, primary care staff, and village, town and county leaders for their opinions on the development of the intervention programme.

Contributors

PZ and JW conceived the project. JX, ML, YB, YL, JW, FJH, GAM and PZ participated in the design and implementation of the project. JX, BT, ML, YB, WY, XZ, ZX, JH, DJ, JS, YL, JW, FJH, GAM and PZ facilitated patient and public involvement and were responsible for setting up the study at each site. BT and JX wrote the first draft of the manuscript and contributed equally to this paper. All authors contributed to the refinement of the study protocol and approved the final manuscript.

Funding

This work is supported by the National Institute of Health Research (NIHR) (NIHR Global Health Research Unit Action on Salt China at Queen Mary University of London) using Official Development Assistance (ODA) funding (16/136/77). The

For peer review only

views expressed in this publication are those of the author(s) and not necessarily those of the NIHR or the Department of Health and Social Care.

Competing interests

FJH is a member of the Consensus Action on Salt & Health (CASH) group, a nonprofit charitable organization, and its international branch World Action on Salt & Health (WASH) and does not receive any financial support from CASH or WASH. GAM is the Chairman of Blood Pressure UK (BPUK), Chairman of CASH and Chairman of WASH and does not receive any financial support from any of these organizations. BPUK, CASH and WASH are nonprofit charitable organizations. All other authors have no competing interests to declare.

Dissemination policy

The findings of this study will be disseminated through discussions or presentations at selected conferences, peer-reviewed publications and the general media.

REFERENCES

1 World Health Organization. Reducing risks, promoting healthy life. Geneva, Switzerland: World Health Organisation, 2002. http://www.who.int/whr/2002 (Accessed 13 Jul 2019).

2 Intersalt Cooperative Research Group. Intersalt: an international study of electrolyte excretion and blood pressure. Results for 24hour urinary sodium and potassium excretion. *BMJ* 1988, 297:319-328.

3 He FJ, MacGregor GA. Reducing population salt intake worldwide: from evidence to implementation. *Prog Cardiovasc Dis* 2010, 52:363-382.

4 He J, Gu D, Chen J, et al. Premature deaths attributable to blood pressure in China: a prospective cohort study. *Lancet* 2009, 374:1765-1772.

5 Wu Y, Huxiey R, Li L, et al. Prevalence, awareness, treatment, and control of hypertension in China: data from the China National Nutrition and Health Survey 2002. *Circulation* 2008, 118:2679-2686.

6 Zhou BF, Stamler J, Dennis B, et al.Nutrient intakes of middle-aged men and women in China, Japan, United Kingdom, and United States in the late 1990s:the INTERMAP study. *J Hum Hypertens* 2003, 17:623-630.

7 He FJ, Wu Y, Feng XX, et al. School based education programme to reduce salt intake in children and their families (School-EduSalt): cluster randomised controlled trial. *BMJ* 2015,

For peer review only

350:h770

8 He FJ, Li J, Macgregor GA. Effect of longer term modest salt reduction on blood pressure: cochrane systematic review and meta-analysis of randomised trials. *BMJ* 2013, 346: f1325.

9 Aburto NJ, Ziolkovska A, Hooper L, et al. Effect of lower sodium intake on health: systematic review and meta-analyses. *BMJ* 2013, 346: f1326.

10 He FJ, MacGregor GA. Salt reduction lowers cardiovascular risk: meta-analysis of outcome trials. *Lancet* 2011, 378:380-382.

11 He FJ, Campbell NRC, Ma Y, MacGregor GA, et al. Errors in estimating usual sodium intake by the Kawasaki formula alter its relationship with mortality: implications for public health. *Int J Epidemiol* 2018, 47(6):1784-1795.

12 Bibbins-Domingo K, Chertow GM, Coxson PG, et al. Projected effect of dietary salt reductions on future cardiovascular disease. *N Engl J Med* 2010, 362:590-599.

13 National Institute for Health and Clinical Excellence(NICE). Guidance on the prevention of cardiovascular disease at the population level. http://guidance.nice.org.uk/PH25 (Accessed 13 Jul 2019).

14 World Health Organization. First global ministerial conference on healthy lifestyles and noncommunicable disease control. https://www.who.int/nmh/events/moscow_ncds_2011/en/ (Accessed 13 Jul 2019).

15 Anderson CA, Appel LJ, Okuda N, et al. Dietary sources of sodium in China, Japan, the United Kingdom, and the Untied States, women and men aged 49 to 59 years: the INTERMAP study. *J AM Diet Assoc* 2010, 110:736-745.

16 Xu J, Wang M, Chen Y, et al. Estimation of salt intake by 24-hour urinary sodium excretion: a cross-sectional study in Yantai, China. *BMC Public Health* 2014, 14:136.

17 He FJ, Zhang PH, Li Y, et al. Action on Salt China. *Lancet* 2018,392(10141):7-9.

18 He FJ, Zhang PH, Luo R, et al. An Application-based programme to reinforce and maintain lower salt intake (AppSalt) in schoolchildren and their families in China. *BMJ Open* 2019,9(7): e027793.

19 Chen X, Guo X, Ma J, et al. Urinary sodium or potassium excretion and blood pressure in adults of Shandong province, China: preliminary results of the SMASH project. *J Am Soc Hypertens* 2015, 9(10): 754-762.

20 Zhang J, Sun L, Liu Y, et al. Mobile Device-Based Electronic Data Capture System Used in a Clinical Randomized Controlled Trial: Advantages and Challenges. *J Med Internet Res* 2017, 19(3):e66.

21 Moore GF, Audrey S, Barker M, et al. Process evaluation of complex interventions:

Medical Research Council guidance. BMJ, 2015, 350:h1258.
